# Machine Learning-Based Modeling of Spatio-Temporally Varying Responses of Rainfed Corn Yield to Climate, Soil, and Management in the U.S. Corn Belt

**DOI:** 10.3389/frai.2021.647999

**Published:** 2021-05-28

**Authors:** Tianfang Xu, Kaiyu Guan, Bin Peng, Shiqi Wei, Lei Zhao

**Affiliations:** ^1^School of Sustainable Engineering and the Built Environment, Arizona State University, Tempe, AZ, United States; ^2^College of Agriculture, Consumer, and Environmental Sciences, University of Illinois at Urbana-Champaign, Champaign, IL, United States; ^3^National Center of Supercomputing Applications, University of Illinois at Urbana-Champaign, Champaign, IL, United States; ^4^Department of Civil and Environmental Engineering, University of Illinois at Urbana-Champaign, Champaign, IL, United States

**Keywords:** corn yield, US Corn Belt, vapor pressure deficit, soil properties, machine learning, random forest, LASSO

## Abstract

Better understanding the variabilities in crop yield and production is critical to assessing the vulnerability and resilience of food production systems. Both environmental (climatic and edaphic) conditions and management factors affect the variabilities of crop yield. In this study, we conducted a comprehensive data-driven analysis in the U.S. Corn Belt to understand and model how rainfed corn yield is affected by climate variability and extremes, soil properties (soil available water capacity, soil organic matter), and management practices (planting date and fertilizer applications). Exploratory data analyses revealed that corn yield responds non-linearly to temperature, while the negative vapor pressure deficit (VPD) effect on corn yield is monotonic and more prominent. Higher mean yield and inter-annual yield variability are found associated with high soil available water capacity, while lower inter-annual yield variability is associated with high soil organic matter (SOM). We also identified region-dependent relationships between planting date and yield and a strong correlation between planting date and the April weather condition (temperature and rainfall). Next, we built machine learning models using the random forest and LASSO algorithms, respectively, to predict corn yield with all climatic, soil properties, and management factors. The random forest model achieved a high prediction accuracy for annual yield at county level as early as in July (*R*^2^ = 0.781) and outperformed LASSO. The gained insights from this study lead to improved understanding of how corn yield responds to climate variability and projected change in the U.S. Corn Belt and globally.

## Introduction

Understanding how different factors (e.g., climate, soil, managements) affect crop yield has critical values to scientific research and practical applications. Identifying and quantifying the relationships between crop yield and various factors allows for better ways to close the yield gap, increase yield potentials (Lobell et al., 2009; van Ittersum et al., 2013), and improve the predictive capability of crop yield for both short-run commodity market and long-term climate change adaptation (Schlenker and Roberts, 2009; Cai et al., 2017; Peng et al., 2018; Li et al., 2019; Kang et al., 2020). The U.S. Corn Belt produces about ~30% of the total global corn production and plays the most critical role in the global corn export. Thus, understanding drivers and improving prediction capability of corn yield variability for the U.S. Corn Belt is critical to forecast the food supply and price fluctuations.

How environment and managements affect corn yield is a classic question that has been studied extensively by agronomists, plant biologists, economists, and recently earth system scientists (Cassman, 1999; Al-Kaisi and Yin, 2003; Kucharik, 2003; Schlenker and Roberts, 2009; Subedi and Ma, 2009; Lobell et al., 2014; Kang et al., 2020). However, there are still a few gaps. Increasingly, more studies have shown that aggregating climate variables into a growing-season condition to predict crop yield is not adequate (Li et al., 2019), as crop has a temporally-varying response to the same climate variable that depends on which phenology stage the crop is in (Butler and Huybers, 2015; Daryanto et al., 2016; Mladenova et al., 2017). Meanwhile, crop yield response to climate may also vary spatially because of the varying planting date and corn maturity length across the large Corn Belt (Zhu et al., 2018). Therefore, regional analyses of crop yield need to explicitly account for the spatial and temporal variabilities in yield response to climate.

Second, though high temperature has been empirically identified as the primary climate variable that affects corn yield in the U.S. (Schlenker and Roberts, 2009; Zhu et al., 2018), the underlying mechanisms still remain less quantified. Specifically, whether temperature affects yield directly through crop growth/phenology or indirectly through the effects of high atmospheric water demands (measured by high vapor pressure deficit, or VPD) that throttles crop stomata, is still debatable. Temperature affects plant physiological processes of photosynthesis and carbon allocation to different components (Kim et al., 2007; Rattalino Edreira and Otegui, 2012; Prasad et al., 2017); high temperature (i.e., “heat stress”) that happens during the reproductive stage of corn leads to reduction in seed number, and high temperature during the grain-filling stage leads to lower seed weight (Rattalino Edreira et al., 2011, 2014; Prasad et al., 2017). High VPD, as an indicator of atmospheric dryness, increases water loss from plants or soil to the atmosphere. Plants respond to high VPD by closing their stomata to avoid faster water loss with a consequence of lowering photosynthesis rate (Grossiord et al., 2020; Kimm et al., 2020). High VPD may also cause faster depletion of soil moisture storage, which may result in more later-season soil moisture deficit (Zhou et al., 2019). Because VPD and temperature are highly correlated, attributing the effects of VPD and temperature on corn yield is thus a critical challenge.

Third, soil properties such as soil available water capacity (AWC) and soil organic matter (SOM) have been identified as two major soil properties that can affect yield (Kravchenko and Bullock, 2000). High AWC allows for a higher water storage in the soil column and therefore more available water for plant to use to alleviate drought stress. High SOM is often associated with high AWC and provides nutrients-rich soil conditions which are conducive for crop growth. When fertilizer is over-applied, the relationship between SOM and corn yield may be confounded. However, heavy rainfall events and the subsequent nutrient leaching may cause nitrogen deficit in the later growing season (Li et al., 2019), and in these cases high SOM can serve as a buffer to reduce yield loss.

Fourth, management practices of farmers, such as those related to farm financial planning and logistics, also affect corn yield (Carter et al., 2018). The important decisions that have direct effects on corn yield include planting date and density, amount and timing of fertilizer application, and seed types. Planting date depends on the field working condition that is strongly associated with weather variability (Urban et al., 2015, 2018). Seed maturity group and planting date together determine the length of the crop growing period. Various studies have shown a positive correlation between a longer growing cycle and increased yield (Lauer et al., 1999; Sacks and Kucharik, 2011; Lobell et al., 2014). Earlier planting can increase yield through lengthening the vegetative period and higher leaf area (Nielsen et al., 2002). Several field experiments have investigated the planting date impacts on corn yield in the Corn Belt, however their findings are region-dependent (Lauer et al., 1999; Nielsen et al., 2002; Van Roekel and Coulter, 2011). In addition, fertilizer amount/timing and cultivar types are all critical for corn growth (Scharf et al., 2002). A comprehensive and quantitative analysis of how these management practices affect yield at broader spatial scales is still missing.

Meanwhile, the increasing availability and accessibility of nationwide datasets of climate, crop survey and management practices in the U.S. provides the opportunity to re-examine the classic question of what affects crop yield at large spatial and temporal scales. In particular, these datasets enable the use of machine learning approaches to infer the relationships between yield and various factors inductively, independent of assumptions and local data typically involved in physical crop models.

In the current study, we synthesize various datasets including climate, soil, management (fertilizer use and planting date) and corn yield data at the county level in the U.S. Corn Belt from 2000 to 2012. Using a data-driven approach, we aim to (1) identify the key drivers of the spatio-temporal variability of rainfed corn yield, and (2) develop machine learning models to predict yield using these drivers and quantify the in-season predictability of rainfed corn yield. To achieve the first objective, we will perform exploratory data analysis to examine the relationships between climate, soil, and management factors and the spatio-temporal variability of rainfed corn yield. The gained insights are then used to develop machine learning models for yield prediction (the second objective) and interpret the learned models. More specifically, we will develop machine learning forecast models to predict annual corn yield and assess the prediction accuracy with varying leading time. As such, the current study will demonstrate the contribution of the data-driven approach and machine-learning to improve the understanding and prediction capability of corn yield response to various factors.

## Materials and Methods

### Data Used and Spatial Division Based on Averaged Climate

**Figure 1:**
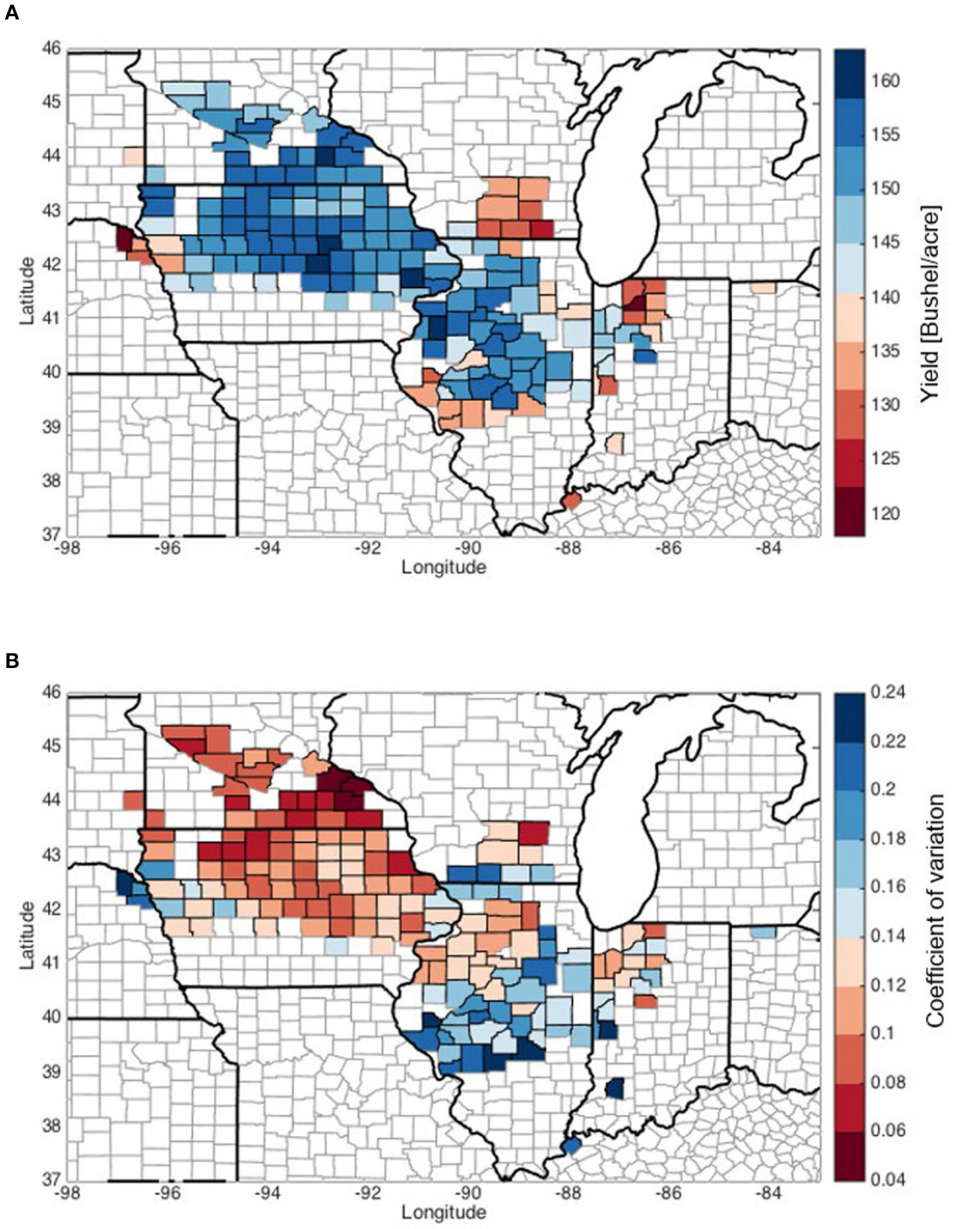
The multi-year average yield and inter-annual coefficient of variation (C.V.), evaluated during 2000–2012. **(A)** Average yield. **(B)** Coefficient of variation.

This study includes data of climate, soil, management (fertilizer use and planting date), and corn yield data at the county level in the U.S. Corn Belt for 2000–2012 (Table 1). Our analysis is based on 2000–2012 county-level annual crop yield data from the National Agricultural Statistics Service (NASS) of the U.S. Department of Agriculture (USDA). In this study, we focus on counties in the U.S. Corn Belt that (1) have zero irrigation acreage (i.e., rainfed), (2) corn planting area exceeds 20% of the total area in that county, and (3) corn planting area exceeds that of soybeans at least by 5% of the county area. Excluding counties with incomplete data record, in total we perform analysis on 166 counties from nine states (Minnesota, South Dakota, Iowa, Nebraska, Wisconsin, Illinois, Indiana, Ohio, and Kentucky). Figure 1 shows the multi-year average of rainfed corn yield and its inter-annual coefficient of variation (C.V.) from 2000 to 2012. Counties in central Illinois exhibit high C.V. due to substantial loss from 2012 drought. Yield average and C.V. from 2000 to 2011, which are more representative for normal conditions, are shown in Supplementary Figure 1.

**Table 1:** Data used in this study.

**Group**	**Variable**	**Notation**	**Unit**
Agronomy	Corn yield	y	bushel/acre
Climate	Monthly total precipitation	P	mm
	Number of dry days	N_d_	–
	Monthly averaged daily mean temperature	T	°C
	Heatwave	N_h_	–
	Monthly averaged daily maximum vapor pressure deficit	VPD	hPa
Soil properties	Available water capacity	AWC	–
	Soil organic matter	SOM	% weight
Management	Planting date (day of year)	DoP	–
	Fertilizer (N) application	N	kg/acre

*Corn yield is the response variable, and all other variables are predictors (explanatory variables)*.

We use the climate record from PRISM (Parameter-elevation Relationships on Independent Slopes Model) (Daly et al., 2008), which interpolates the daily observations from over 13,000 stations across the conterminous U.S. into grids at 4-km resolution based on digital elevation and empirical model. Specifically, daily precipitation, mean temperature, and maximum VPD from PRISM data are used to derive metrics for climate condition and climate extreme events, which are described in detail in section Feature Selection and Correlation Analyses Between Climate Variables and Yield. Soil variables consist of AWC and SOM based on the gridded Soil Survey Geographic (gSSURGO) dataset, which has a spatial resolution of 30 m (NRCS, 2016). The dataset is a compilation of data collected through field survey and sampling campaigns conducted by the USDA NRCS. Expressed as a volume fraction, AWC describes the amount of water soil can store that is available to plants. It is commonly estimated as the difference between the water contents at 1/10 or 1/3 bar (field capacity) and 15 bars (permanent wilting point) tension and adjusted for salinity, and fragments. SOM is the fraction of the soil that consists of plant or animal tissue; organic matter contributes to soil fertility. The climate variables are aggregated to county level by taking the average spatially. The soil variables are averaged to county level by using the 2016 National Land Cover Database (NLCD) as a mask of cropland (Wickham et al., 2014). In addition, the analysis considers two management variables: planting date and nitrogen fertilizer application amount. Planting date is averaged over each county from the randomly resampled 100 corn fields that are based on the Risk Management Agency (RMA) dataset of USDA (Lobell et al., 2014). We used county-wise nitrogen fertilizer application estimates from the NUGIS dataset [International Plant Nutrition Institute (IPNI), 2011], which is based on the nitrogen fertilizer sale information aggregated to the county level, assuming locally sold fertilizer is used locally [International Plant Nutrition Institute (IPNI), 2011].

**Figure 2:**
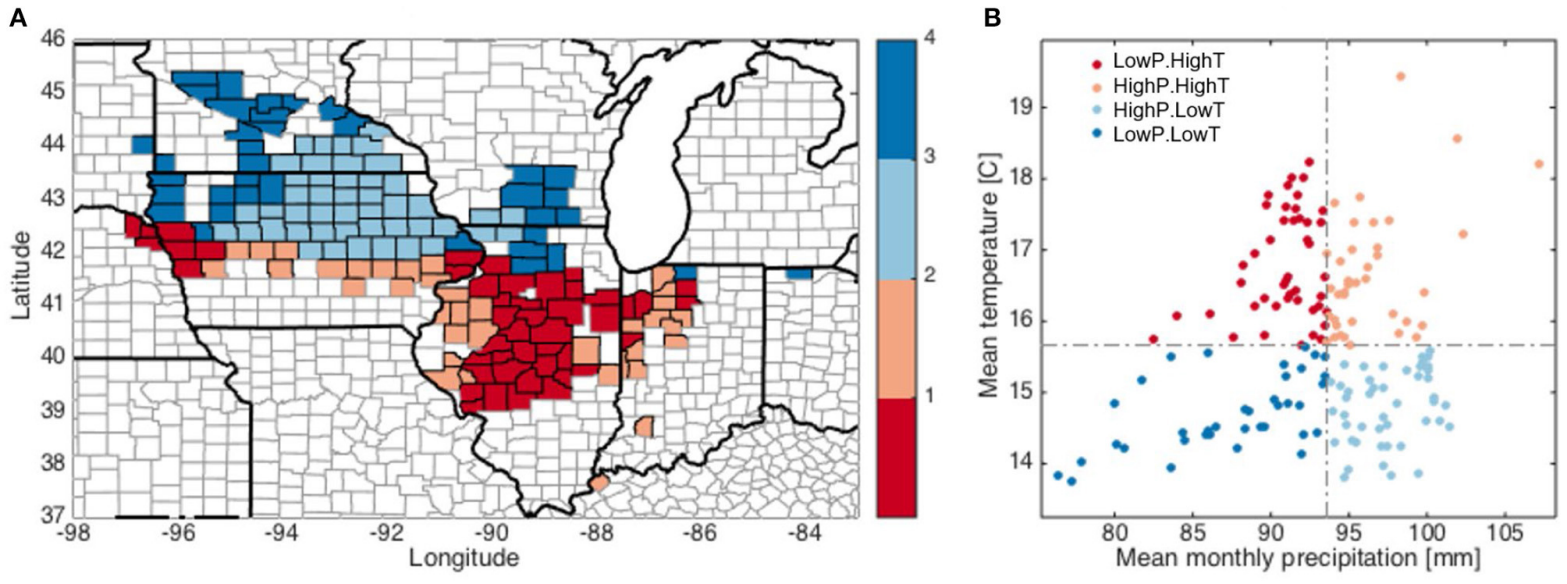
Dividing the counties in this study into four groups based on the growing season average temperature and monthly precipitation (from March to September) based on climatology during 1980–2012 **(A)**. The dashed lines in **(B)** indicate dividing criteria.

To better understand the varying patterns of corn yield response across different climate conditions, we divide the 166 counties into four groups according to their long-term average of precipitation and mean temperature from March to September. Climate conditions in March and April are included because pre-growing season soil moisture also affects crop yield (Li et al., 2019). Four types of climate are defined: HighP.HighT, HighP.LowT, LowP.HighT, and LowP.LowT (Figure 2A). The dividing criteria are shown in Figure 2B and are selected as the median of the countywise monthly precipitation (P) and temperature (T), respectively, averaged over March to September from 1980 to 2012. It is worth noting that high or low P (or T) are defined in relative sense, as our study area (the core rainfed part of the U.S. Corn Belt) in general has sufficient rainfall and agreeable temperature for crop growth. Overall, counties having latitude above 42N are grouped into the low T climates, while precipitation pattern is less obvious. The HighP.LowT group is primarily comprised of counties in southern Minnesota and northeastern Iowa, while LowP.LowT counties are scattered in Minnesota, western Iowa, southern Wisconsin and northern Illinois. The HighP.HighT group includes counties scattered in Iowa, Illinois and Indiana. Lastly, LowP.HighT counties are mainly located in western Iowa and central Illinois. Performing analyses for each group enables investigating how the crop yield response varies spatially and across the climate gradient.

### Feature Selection and Correlation Analyses Between Climate Variables and Yield

Selecting which climate variables, including which one and at what time, to use as inputs is an important step for estimating/predicting crop yield. Besides the commonly-used climate variables P, T, and VPD, we also use the PRISM data to calculate the following climate extreme metrics for each month, based on the WMO CLIMDEX climate extremes indices (Karl and Easterling, 1999; Donat et al., 2013). They are: (1) dry-spell (maximum number of consecutive days without precipitation), (2) the number of days without precipitation, (3) wet-spell (maximum number of consecutive days for which precipitation exceeds daily climatology by over 3 mm), (4) accumulated precipitation exceeding monthly climatology, (5) maximum consecutive 5-day precipitation, (6) precipitation intensity index (i.e., average precipitation amount on rainy days), (7) heatwave (maximum consecutive number of days for which the daily maximum temperature exceeds the monthly climatology by 5°C), (8) the number of days for which the daily maximum temperature exceeds 30°C, (9) average value of daily temperature range, (10) cold-wave (maximum consecutive number of days for which the daily minimum temperature is below the monthly climatology by over 4°C), and (11) extreme heat days (i.e., accumulated degree^*^day exceeding 30°C).

We then perform correlation analysis (results not shown due to space limit) to determine which climate metrics we will include in later modeling analysis. Specifically, we examine the degree of correlation among the variables corresponding to each month, as well as correlation between yield and the variables of each month during the growing season. Co-linearity is detected among many of the variables. For example, the monthly averaged Tmax, Tmin, and Tmean are strongly correlated with each other at any month. Correlation analyses suggest Tmax has overall the highest correlation with yield. Similarly, among the WMO CLIMDEX climate extreme indices the number of dry days (denoted as N_d_) and heatwave (denoted as N_h_) yield overall the highest correlation with yield. In order to reduce redundancy and construct a parsimonious dataset for the downstream analysis, we include monthly total precipitation (P), monthly averaged daily Tmean (T), monthly averaged daily maximum VPD, Nd, and Nh (Table 1) for the correlation analyses and data-driven modeling results presented in section Results. Inclusion of the variables that were not selected does not significantly improve the prediction accuracy of yield. We first detrend the annual crop yield, total P and mean T via linear regression. Next, for each year we multiply daily P (T) with the ratio of detrended annual P (T) to annual P(T) before detrending. The resulting detrended daily P and T are then used to calculate the monthly P, T, N_d_, and N_h_. All variables are scaled to [0, 1].

Besides the climate indices, we also consider two soil property variables [soil available water content (AWC) and soil organic matter (SOM)], and two management practice variables [county-averaged planting date (DoP) and nitrogen fertilizer application (N)] (Table 1).

### Using Machine Learning Models to Predict Rainfed Corn Yield

Correlation analyses, as a useful way of data exploratory analysis, provide a comprehensive view on the empirical relationships between corn yield and individual predictors. We then use two widely used parametric and nonparametric machine learning algorithms, random forest and Least Absolute Shrinkage and Selection Operator (LASSO), respectively, to predict rainfed corn yield using the predictors (input variables) in Table 1.

#### Random Forest

Random Forests (Breiman, 2001) are a non-parametric, non-linear ensemble machine learning algorithm. A random forest model (RF) is comprised of *N* decision trees and outputs the mean prediction of individual trees. Each tree is trained using a bootstrap sample of the dataset {**x**_**i**_, *y*_*i*_}, *i* = 1, …, *n*, where ${\text{x}}_{i}={\left\{x_{i1},...,x_{ij},...,x_{ip}\right\}}^{T}$ is a vector comprised of *p* input variables and *y*_*i*_ is the corresponding output (i.e., corn yield). The tree recursively partitions the input space into rectangular regions through a sequence of binary splits. A binary split at *X*_*j*_ = *t* partitions the input space into two regions and fit a constant value to each region (*c*_1_, *c*_2_, respectively). The best splitting variable *X*_*j*_, split point *s*, and *c*_1_, *c*_2_ are found by minimizing the error sum of squares. At each split during the construction of a single tree, the RF algorithm identifies the best splitting variable from a randomly selected subset of input variables. After training, RF makes prediction for a new data point by averaging the predictions from each tree. The RF algorithm also calculates a variable importance score based on the total decrease in node impurities from splitting on the variable, averaged over all trees (Hastie et al., 2001). For regression, the node impurity is measured by error sum of squares.

While a decision tree is highly sensitive to noise in the training data, RF has proven to have better generalization performance because of bootstrap aggregating and using random subset of inputs as candidates for splitting variable selection (Breiman, 2001; Hastie et al., 2001). The candidate splitting variable selection increases the chance that any single variable gets included in a random forest especially for weak inputs. RF has gained popularity in various fields such as meteorology (Cloke and Pappenberger, 2008; He et al., 2016), soil science (Ließ et al., 2012), hydrology (Naghibi et al., 2016; Xu et al., 2017), and remote sensing (Xu et al., 2019).

#### LASSO

Least Absolute Shrinkage and Selection Operator (LASSO) is a widely used linear regression-based method that adds a *L*_1_ penalty term to the ordinary least squares in order to keep the regression coefficients small (Tibshirani, 1996). Because of the *L*_1_ penalty term, LASSO typically sets some of the regression coefficients to zero. The number of zero coefficients depends on a penalty parameter, which is usually selected through cross validation. As such, the algorithm performs a feature selection and has been reported robust for high dimensional regression problems. Because of its good generalization performance, sparsity and interpretability, LASSO has been used in various applications (e.g., Everingham et al., 2009; Vandal et al., 2017; Anda et al., 2018).

#### Quantifying In-season Predictability of Rainfed Corn Yield

We train RF and LASSO models for each climate group and for all counties involved in this study for the period 2000–2012, using as inputs *x*_*i*_ = [*P*_*i*_, *N*_*d,i*_, *T,N*_*h,i*_, *VPD*_*i*_, *DoP*_*i*_, *AWC, SOM, Ni*]. The climate inputs consist of the monthly data from April to August in i-th year, e.g., precipitation *P*_*i*_ = [*P*_*Apr,i*_, *P*_*May,i*_, *P*_*Jun,i*_, *P*_*Jul,i*_, *P*_*Aug,i*_]. The planting date and fertilizer use vary year by year, while soil properties are assumed to be constant in time. Once a RF model is constructed, the algorithm calculates variable importance of climate data, planting date and soil texture to corn yield. In this way, we identify which factors, at which stage, have the highest impacts on yield.

We will also use the RF and LASSO models to investigate the predictability of rainfed corn yield. In particular, we aim to quantify how the predictability of corn yield evolves over the course of the growing season as more data become available. Such analyses will shed light on how long in advance a reliable prediction can be made. For every forecasting stage (month), we use all the data from April to the targeted month as inputs to build a model to predict the end-of-season corn yield. For example, in order to assess the corn yield predictability by the end of June, we build a RF or LASSO model using time-invariant data (i.e., planting date, soil texture) and climate data from April to June. Prediction at a later month will include more climate data. Each time, we randomly split the data points into a training set (67%) and a test set (33%), which is used to assess the prediction performance. More specifically, we calculate the coefficient of determination *R*^2^and root-mean-square error (RMSE) by comparing the RF/LASSO model predicted yield with reported county-wise crop yield (USDA NASS data). The TreeBagger and Lasso library functions in Matlab with default hyperparameters were used to build the models. To reduce the effect of randomness in dataset split and RF training process, every experiment is repeated 20 times, and the average *R*^2^ is reported in the section Results. The prediction performance of RF and LASSO models are compared.

## Results

### Temporally Varying Correlation Patterns Between Climate Variables and Corn Yield Across the Corn Belt

**Figure 3:**
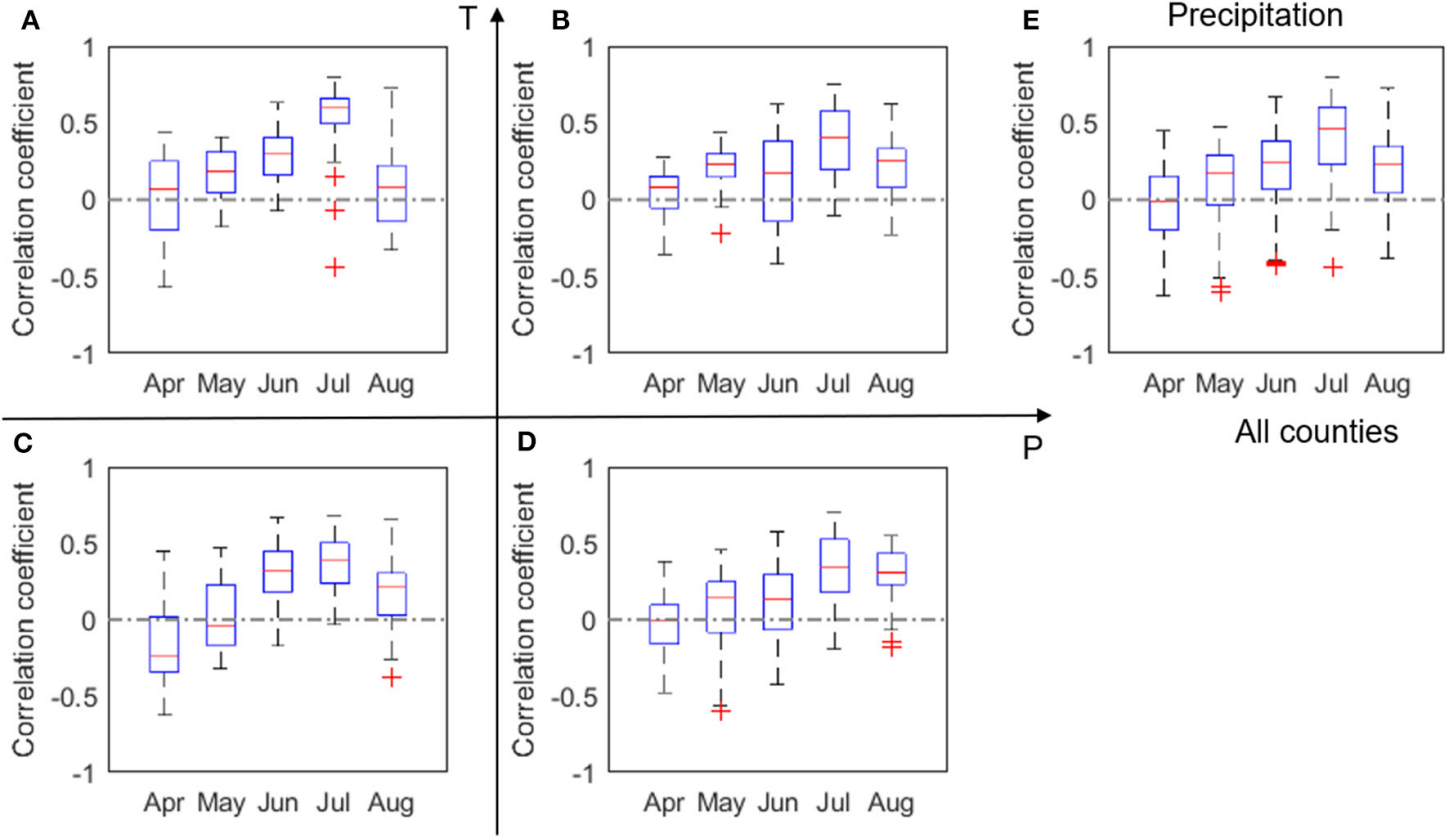
Box plots of correlation coefficient (*r*) between corn yield and monthly total precipitation, P, evaluated for four groups **(A–D)** and all analyzed counties **(E)** in 2000–2012. The red + signs indicate outliers.

**Figure 4:**
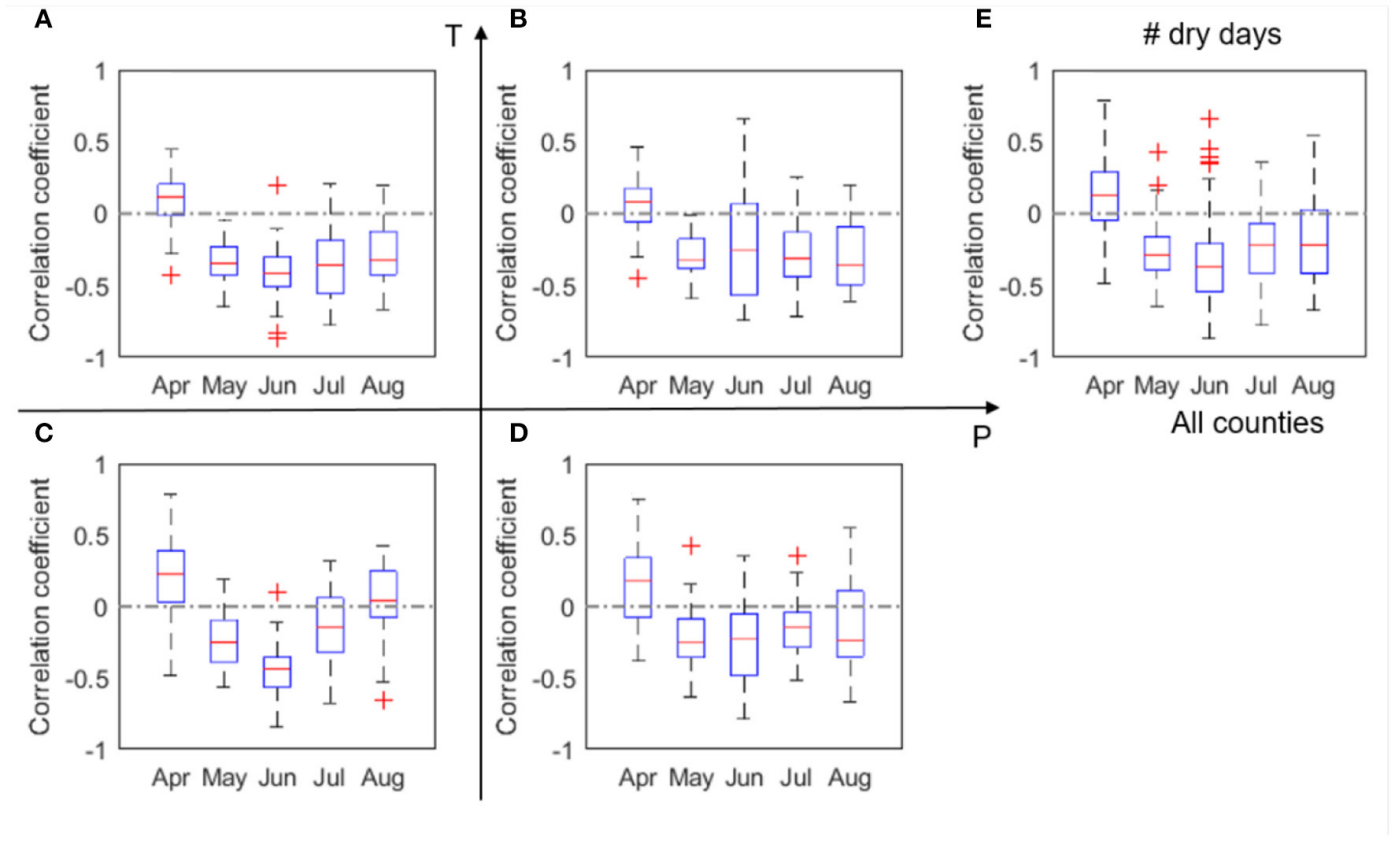
Box plots of correlation coefficient (*r*) between corn yield and number of dry days, N_d_, evaluated for four climate groups **(A–D)** and all analyzed counties **(E)** in 2000–2012. The red + signs indicate outliers.

**Figure 5:**
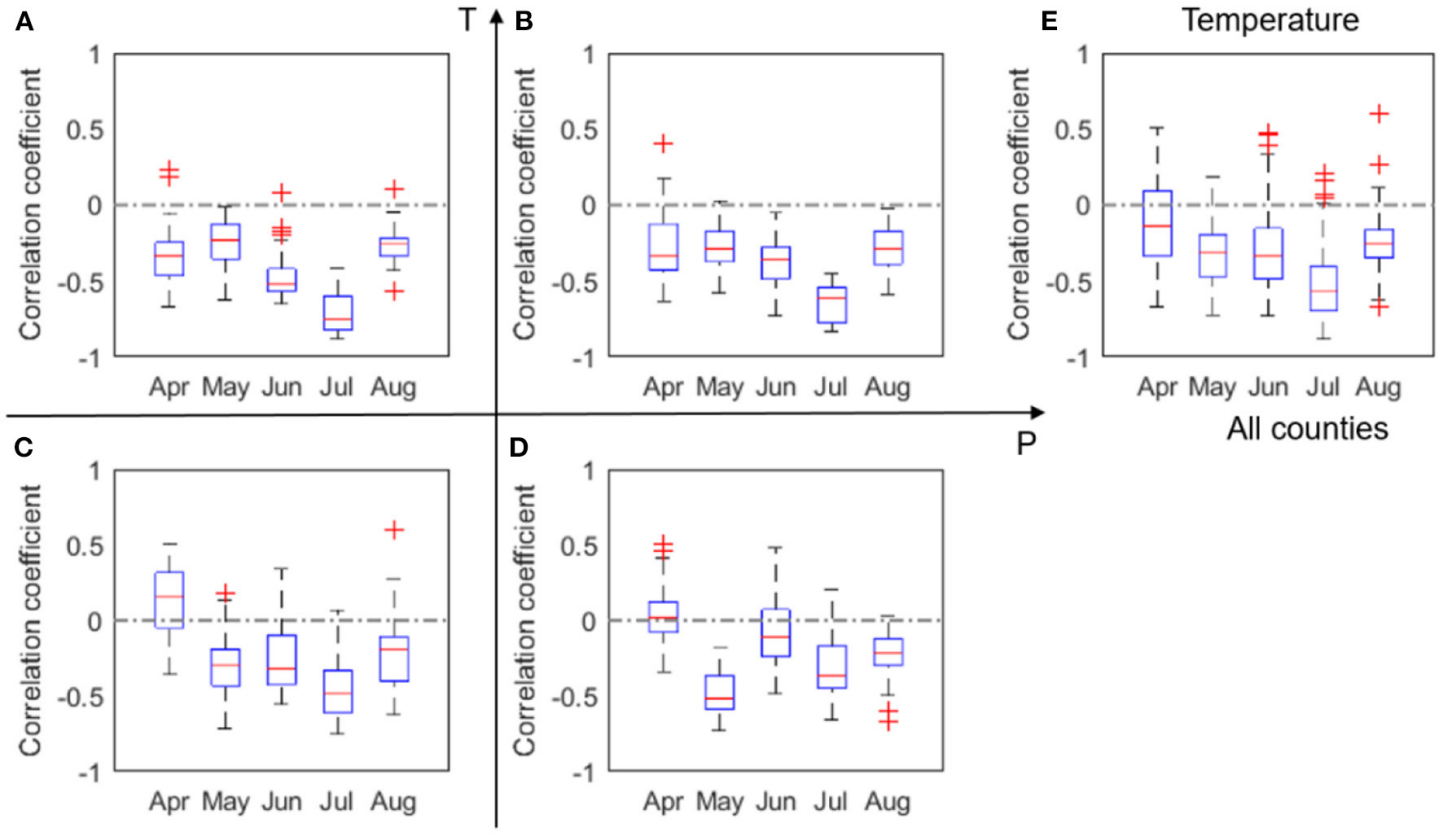
Box plots of correlation coefficient (*r*) between corn yield and monthly mean temperature, T, evaluated for four groups **(A–D)** and all analyzed counties **(E)** in 2000–2012. The red + signs indicate outliers.

**Figure 6:**
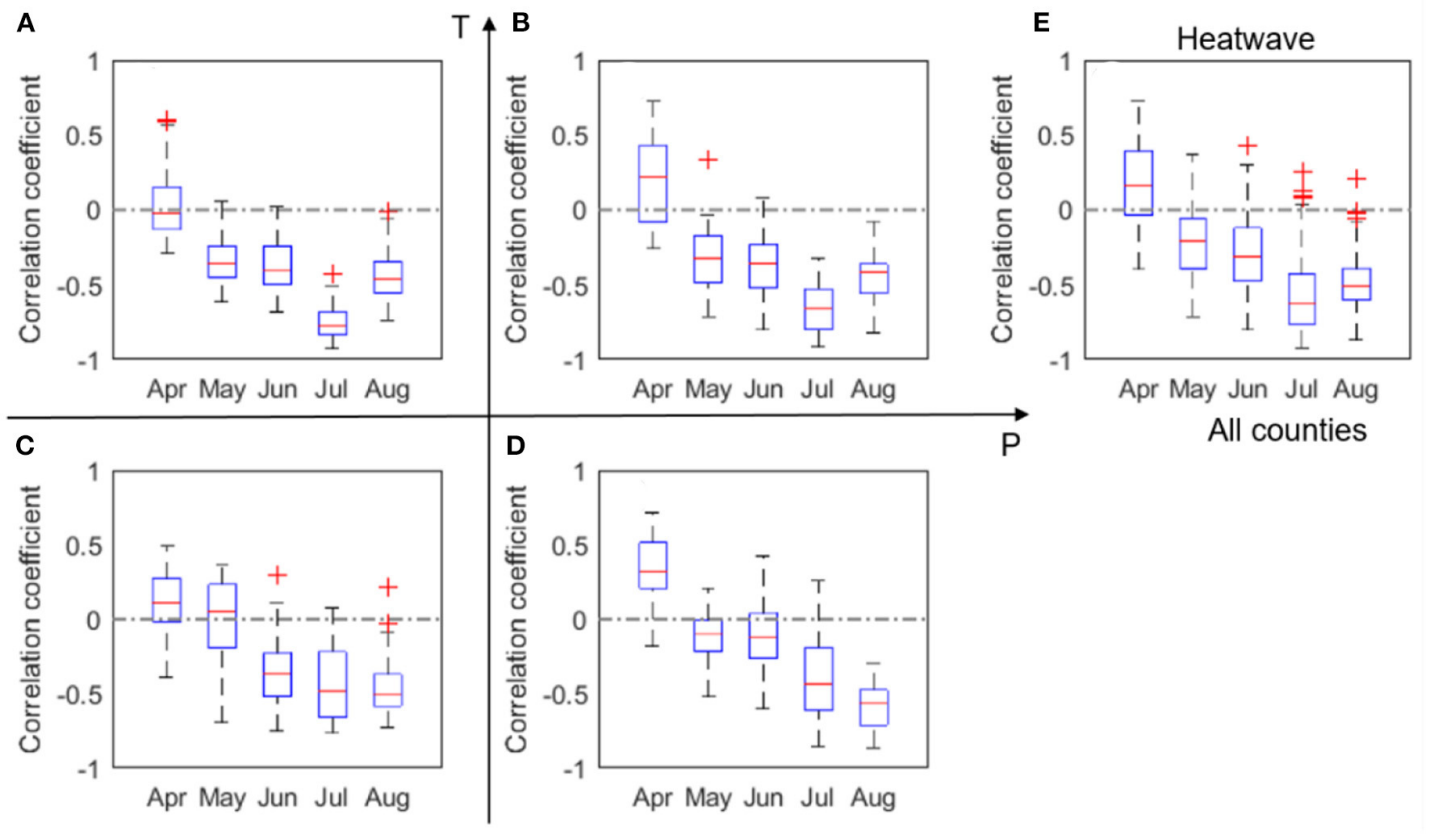
Box plots of correlation coefficient (*r*) between maize yield and heatwave, N_h_, evaluated for four groups **(A–D)** and all analyzed counties **(E)** in 2000–2012. The red + signs indicate outliers.

**Figure 7:**
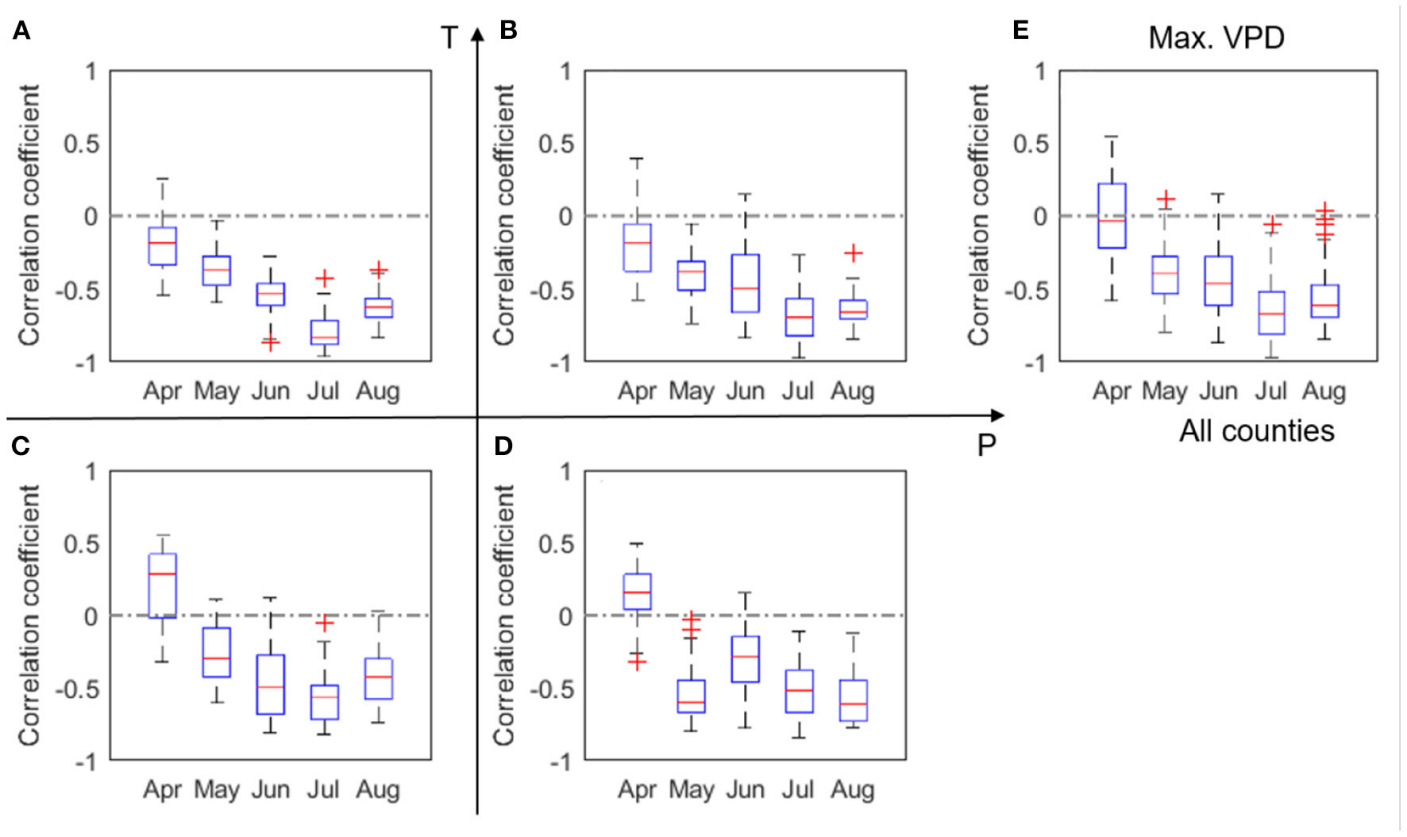
Box plots of correlation coefficient (*r*) between corn yield and VPD, evaluated for four groups **(A–D)** and all analyzed counties **(E)** in 2000–2012. The red + signs indicate outliers.

The correlation coefficients (*r*) between corn yield and monthly P, number of dry days, T, heatwave, and VPD, respectively, are shown in Figures 3–7. Similar plots, but with data 2000–2011, can be found in Supplementary Figures 2–6. Because of the substantial loss in the 2012 drought, low to moderate differences are noticeable between Figures 3–7 and Supplementary Figures 2–7. In order to consider the whole spectrum of variability including drought conditions, our discussion here is focused on the whole dataset of 2000–2012.

We found a clear positive correlation between corn yield and July precipitation, especially in the LowP.HighT climate type (Figure 3). This is as expected, because water stress in the critical month of July is expected to negatively affect corn yield (Çakir, 2004; Li et al., 2019). The positive correlation is more evident in the LowP.HighT group (Figure 3A) than other groups, as precipitation is the limiting factor in this climate. Correlation in the LowP.LowT climate type is overall weaker than the LowP.HighT group. For HighP.LowT group, strong positive correlation can also be observed for August precipitation. This is because the growing stage is usually delayed in cold regions. Meanwhile, Figure 4 shows the correlation between yield and monthly number of dry days. While July is the critical month in terms of monthly total precipitation (Figure 3), number of dry days in June has the most significant negative correlation with yield for the drier climates. A high value of N_d_ in June results in water stress in the vegetative stage and thus causes reduction in yield.

We also found an evident negative correlation between corn yield and mean temperature particularly in July. This negative correlation is the strongest for the hot and arid climate and slightly weaker for the HighP.HighT group, because higher precipitation alleviates water stress and in general cools the plant through more evapotranspiration. However, water supply cannot fully offset high water demand due to high temperature in June and July. The negative correlation is less evident for the HighP.LowT group. Under this climate condition, water supply may be sufficient to offset water demand. Figure 5 also shows that April temperature is negatively correlated with yield for hotter climates, but not for colder climates. As explained in more detail in section Effect of Planting Date, this spatial distinction is likely caused by planting date. For states that have warmer climates, we find that higher April temperature is related to early planting date, which affects yield.

Figure 6 shows that negative correlation between yield and heatwave is evident in June, July and August for most counties. The most negative correlations between heatwave and yield show in July for most climates except the HighP.LowT group. This climate group covers the northern part of the study area and has a delayed growing season. It is also worth noting that the impacts of heatwave show some difference with the impacts of temperature (Figure 5), which may lend support to earlier modeling work that shows adding heat-stress specific features in modeling (both process-based and statistical models) can in general better capture crop yield variability (Gabaldón-Leal et al., 2016; Jin et al., 2016).

The correlations between VPD and yield (Figure 7) bears similarity to the correlation pattern between temperature and yield (Figure 5). This similarity is largely due to the high correlation between VPD and temperature, which also confirms the challenges of attributing the impacts of VPD and temperature on crop yield. The absolute values of the correlation coefficients in Figure 7 are generally higher than those in Figure 5, indicating the VPD impacts on crop yield may be stronger than the temperature impacts. The next section provides a more detailed analysis on this observation.

### Disentangling the Confounding Effects of Temperature and VPD on Yield

**Figure 8:**
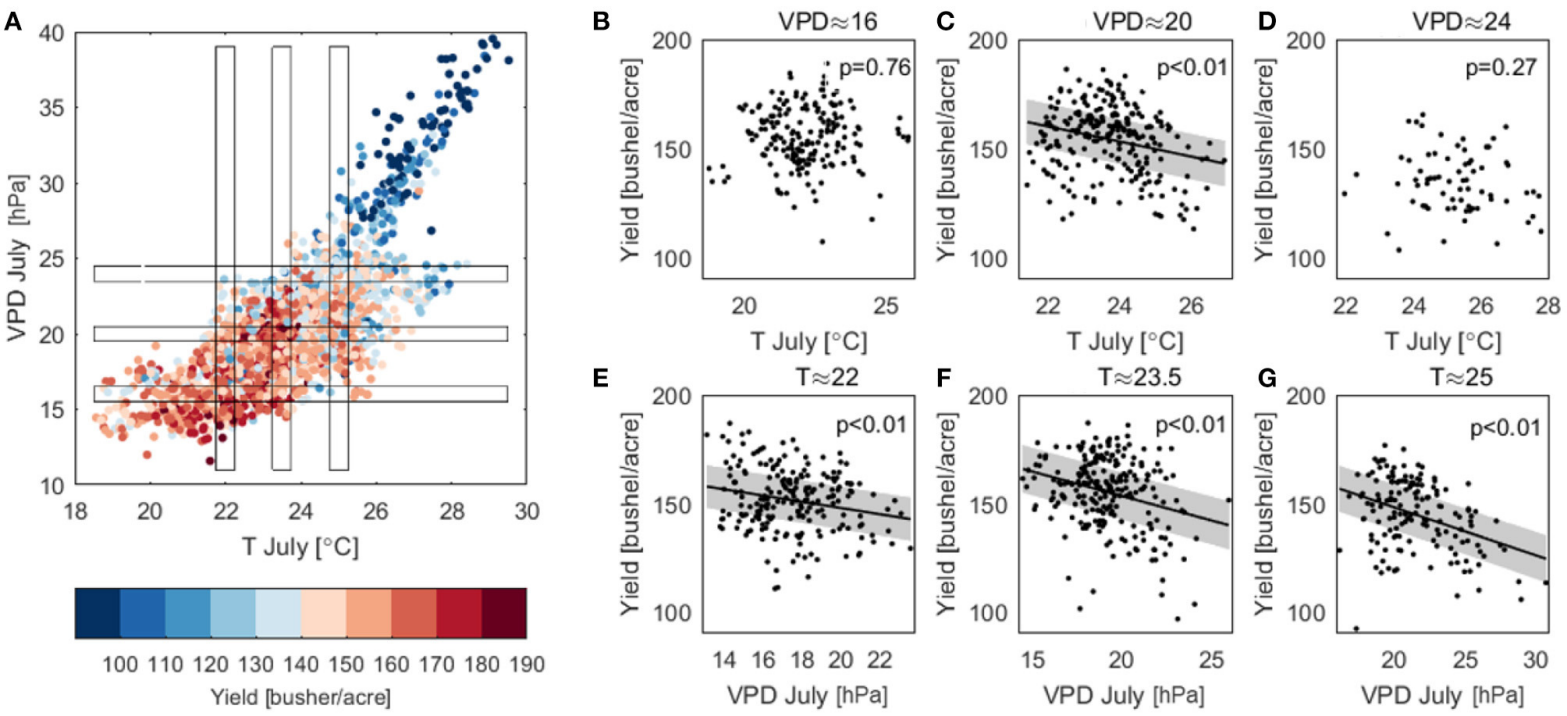
**(A)** Color-encoded yield relation with July mean temperature and maximum VPD. **(B–D)** Scatter plot of yield vs. July mean temperature when maximum VPD is fixed within a range of ±0.5. **(E–G)** Scatter plot of yield vs. July maximum VPD when mean temperature is fixed within a range of ±0.25. Also shown are linear regression with 95% confidence interval reported with the *p*-value of the linear regression slope. Linear regression is not plotted when the linear trend is not significant.

July T and VPD are correlated with each other (ρ = 0.77, Supplementary Figure 10) and both highly correlated with crop yield (Figures 5, 7). Here we attempt to disentangle the specific contributions of T and VPD on corn yield using a parsimonious approach. We plot all the county-year observations of July T and VPD as a scatterplot in Figure 8A, colored by the value of corn yield. We then examine how yield changes with VPD when T is fixed by focusing on a narrow range of T. We repeat the same analysis to examine the trend of corn yield change with T when VPD is fixed within a small range. Figures 8E–G shows that when T is fixed, corn yield reduces sharply with increased VPD; in contrast, when VPD is fixed, the declining trend of corn yield as T increases is less evident (Figures 8B–D).

To further investigate how VPD and temperature affect crop yield, using least squares regression we fit a quadratic polynomial function to the data shown in Figure 8A, with *R*^2^ of 0.54 and RMSE of 14.8 bushel per acre:

(1) $$\begin{array}{l} \text{Yield}=-256+44.6\text{T}-9.1\text{VPD}-1.3{\text{T}}^{2}+0.8\text{T}\cdot \text{VPD}-0.30\text{VP}{\text{D}}^{2}. \end{array}$$

**Figure 9:**
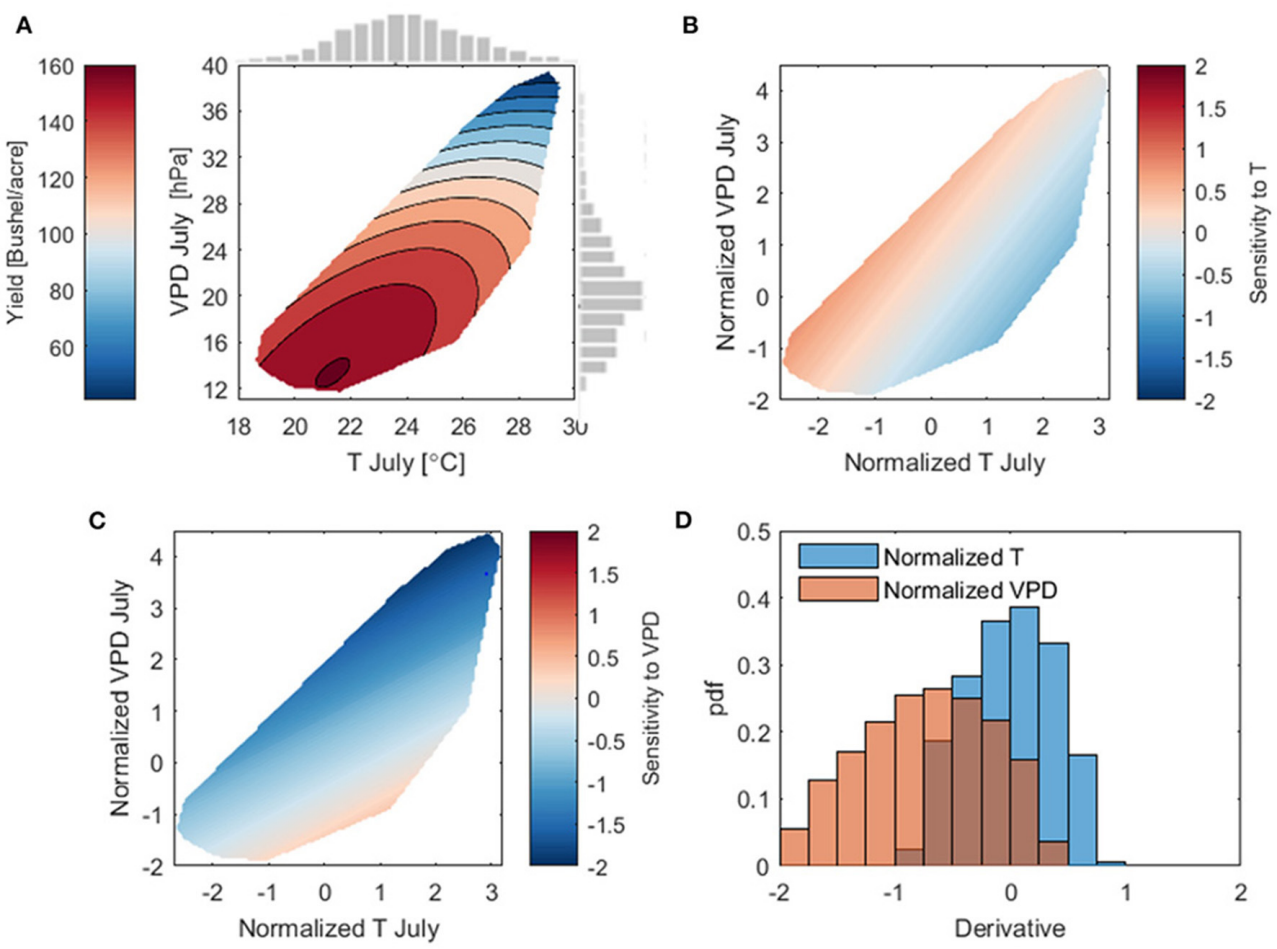
**(A)** The contour plots of fitted surface given by Equation (1); the gray bars show histogram of July mean temperature and maximum VPD. Uncertainty of the regression is reported in Supplementary Figure 7. **(B)** The derivative of fitted yield to T_Jul_, calculated from Equation (1). **(C)** The derivative of fitted yield to VPD_Jul_. In **(B,C)**, both T and VPD are normalized to have the zero mean and unit standard deviation, so that they can be inter-compared. **(D)** The probability distributions of the derivatives of yield to the normalized T_Jul_ and VPD_Jul_.

The fitted surface is shown in Figure 9A, and half width of 90% prediction interval is shown in Supplementary Figure 7. To quantitatively assess the change of corn yield with VPD at fixed T, we calculate the derivative of yield to VPD in Equation (1); similarly, the derivative of yield to T from Equation (1) indicates the yield sensitivity with T for any fixed VPD. The resulting yield sensitivity to T and VPD are plotted in Figures 9B,C, respectively. To fairly compare the sensitivity, we normalized both VPD and T observations to have zero mean and unit standard deviation. We further plot all the sensitivity values in Figures 9B,C as the empirical probability density in Figure 9D.

Figure 9 shows that yield change to the normalized VPD at a fixed T (Figure 9C) is much larger than yield change to the normalized T at a fixed VPD (Figure 9B), suggesting that VPD is the dominant factor in changing corn yield. Furthermore, Figure 9D shows that yield change to VPD is always negative, and in general this change is usually much larger than to T. More interestingly, Figure 9D shows that yield change to T can be both positive and negative, which mirrors the field-level empirical studies of a concave response of photosynthesis to temperature.

**Figure 10:**
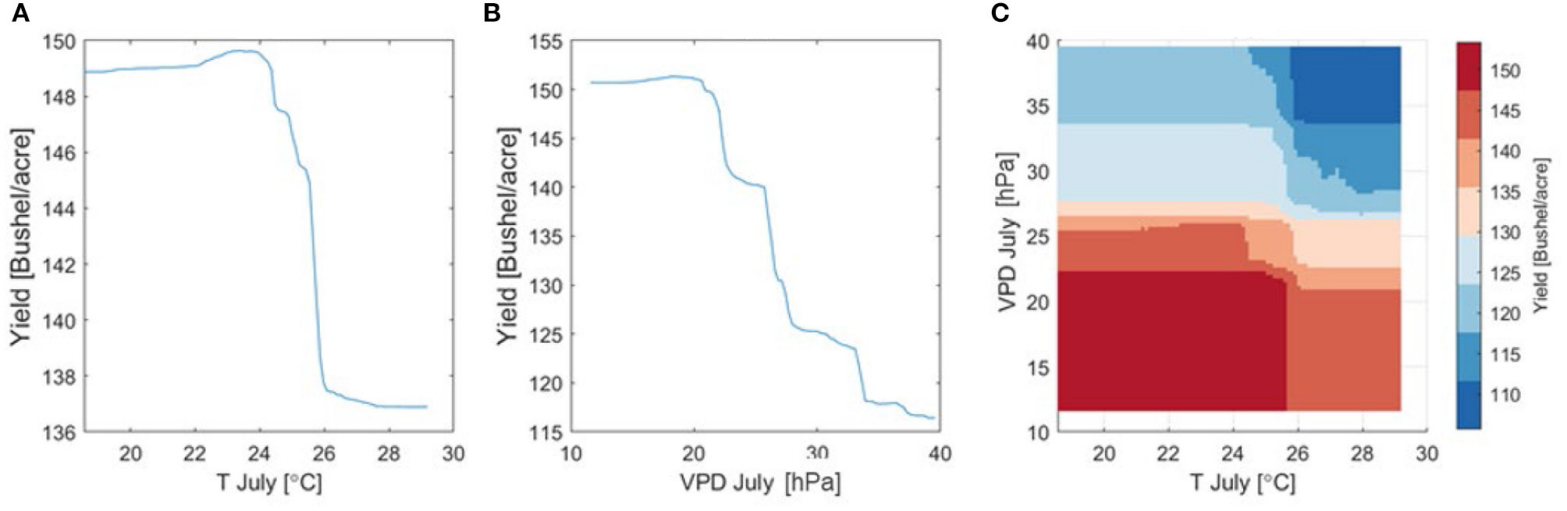
Partial dependence plots based on the trained random forest model using climatic, soil property, and management factors for **(A)** July average daily mean temperature, **(B)** July average daily maximum VPD, and **(C)** both T and VPD in July.

Next, we use the random forest algorithm to investigate nonlinear yield response to July T and VPD that are not captured by the linear regression (Equation 1). To this end, a random forest model is trained using the county level yield and input variables listed in Table 1. We use the trained model to generate partial dependence plots for July T, VPD, and both T and VPD, respectively, as shown in Figure 10. Partial dependence plots show the dependence between the output variable (yield) and one or more of the input variables by marginalizing over the values of all other input variables. They can be interpreted as the expected output as a function of only the selected input variable(s). Figure 10 confirms the findings from Figure 9 and shows a non-monotonic response of yield to increasing July T, which is manifested by a small increase of yield when T < 24° followed by a sharp decrease. This suggests that 24° may be a temperature threshold within the study area.

### Effect of Soil Properties on Corn Yield

**Figure 11:**
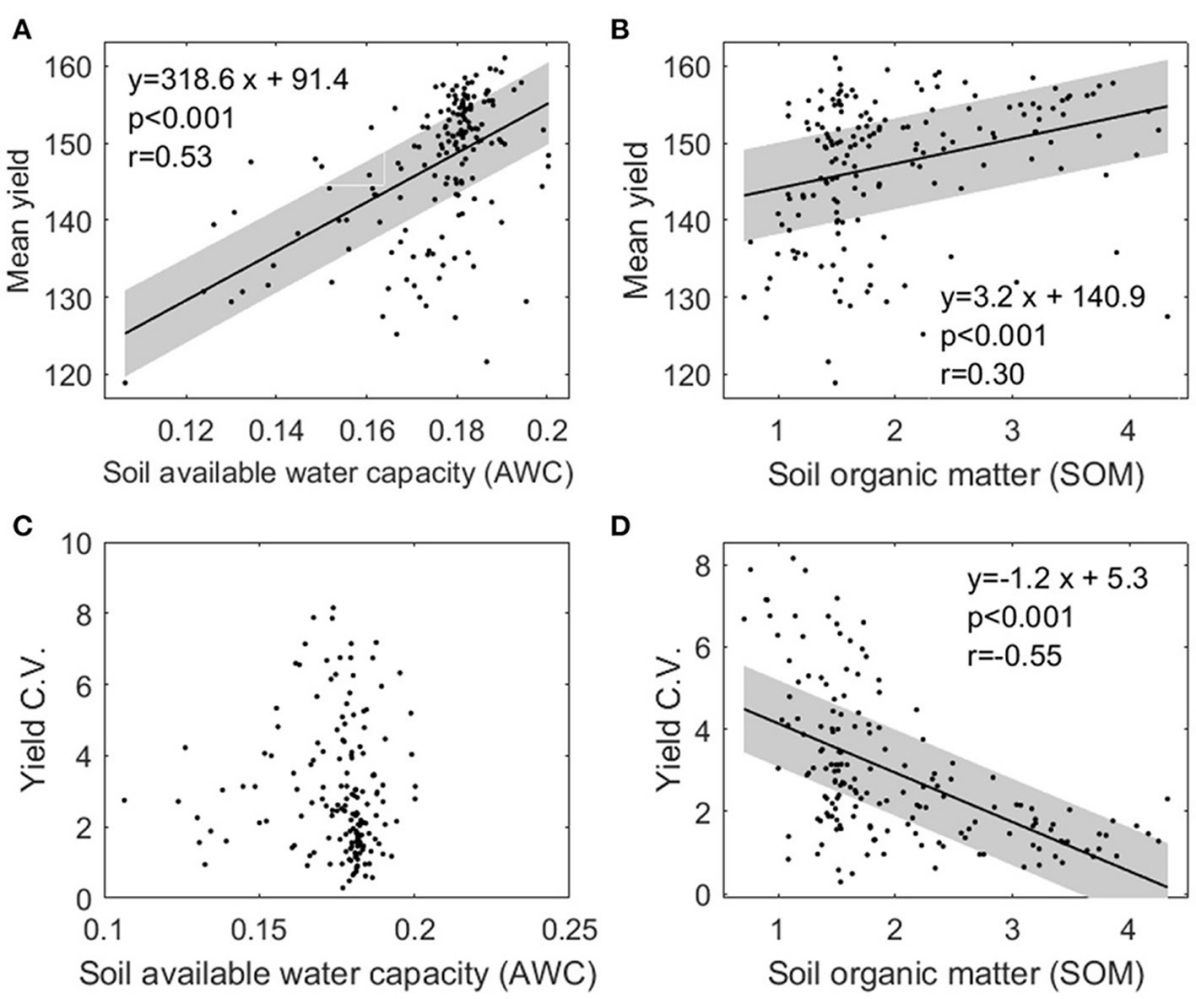
The 13-year yield average and coefficient of variation plotted vs. soil available water content and soil organic matter for each county from 2000 to 2012. Also shown are linear regression with 95% confidence interval, the *p*-value of the linear regression slope, and correlation coefficient (*r*) calculated from data. Linear regression is not plotted when the linear trend is not significant.

Figures 11A,C show that the mean yield increases with the higher soil AWC and SOM. This result is largely expected, as soil that has higher AWC can hold more water during the growth season, and higher SOM indicates favorable soil conditions for crop growth. Figure 11D shows a statistically significant decreasing trend of yield variability as SOM increases, suggesting that higher SOM could buffer yield variability.

### Effects of Management on Corn Yield

#### Effect of Planting Date

**Figure 12:**
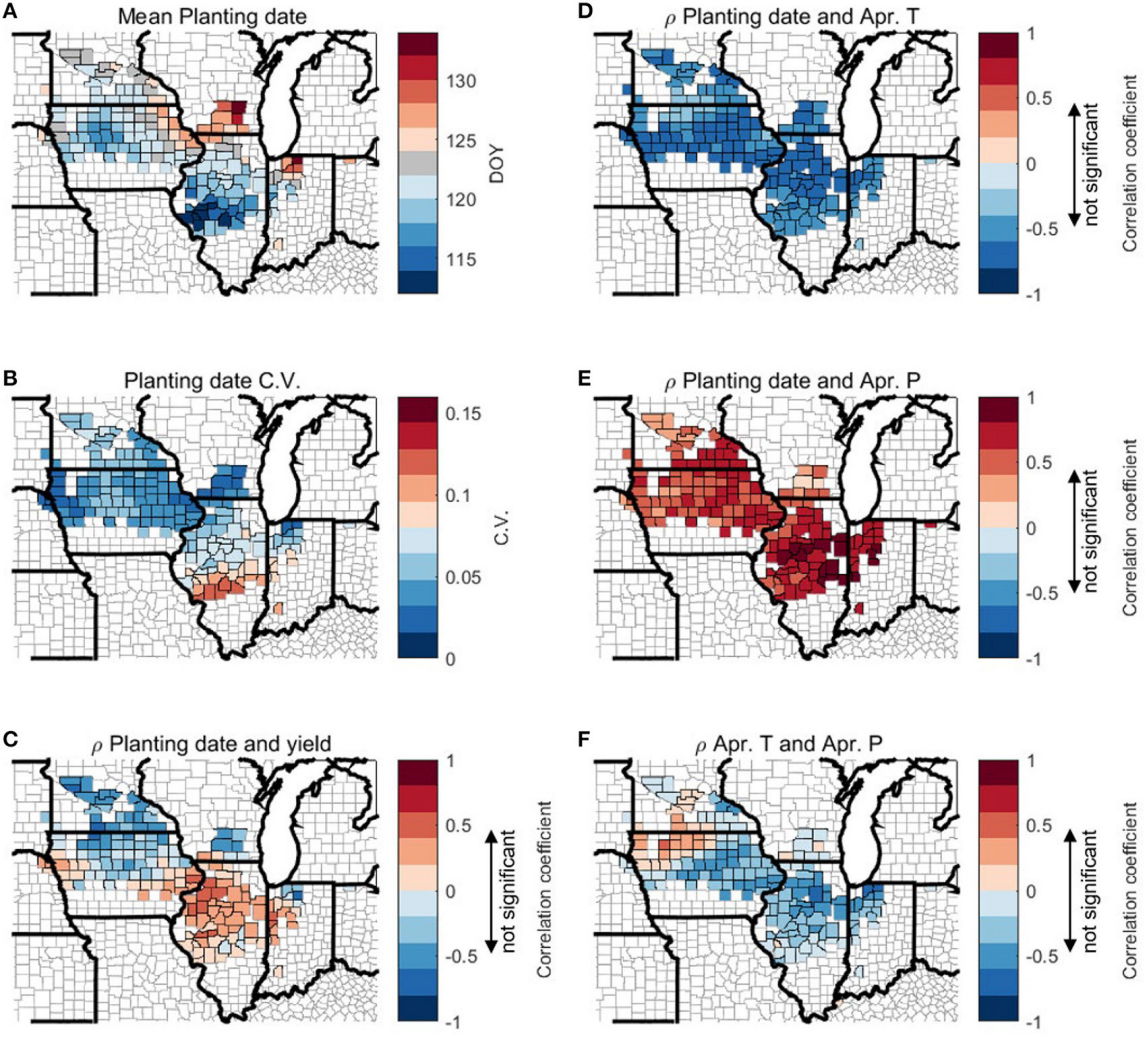
Spatial plot of county-level multi-year mean value **(A)** and coefficient of variation (C.V.) **(B)** of planting date and correlation coefficient (*r*) among planting date, April mean temperature, April total precipitation, and yield **(C–F)**. Range of correlation coefficient that is not significant (*p* > 0.1) is marked on colorbars.

**Figure 13:**
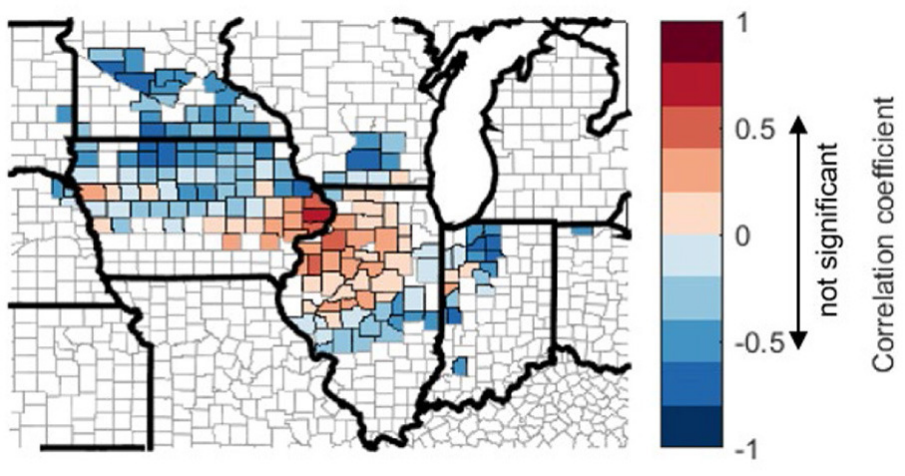
Spatial plot of county-level correlation coefficient (*r*) between planting date and yield from 2000 to 2011. Range of correlation coefficient that is not significant (*p* > 0.1) is marked on colorbar.

Here we examine the effect of planting date on rainfed corn yield. Figure 12A shows the average planting date at the county level, which exhibits a weak north-south gradient with early planting in the south of the study domain and late planting in the north. Figure 12C shows that the correlation between planting date and crop yield varies spatially, with the south part showing a positive correlation (i.e., late planting corresponds to high yield) and the north part showing a negative correlation (i.e., early planting corresponds to high yield). A similar plot for 2000–2011 is shown in Figure 13 in order to account for potential skewness resulting from the 2012 drought. Figure 13 shows similar spatial patterns as in Figure 12C for northern regions. For counties in central Illinois south of 40° and eastern Indiana, excluding 2012 data leads to negative correlation between planting date and yield.

We further explore the inter-annual relationship between planting date and April climate Figure 12. In all the counties, planting is later when April is cooler (Figure 12D) and wetter (Figure 12E). This is consistent with previous studies and anecdotal understanding that cold temperature and heavy rainfall in April postpones planting in the U.S. Corn Belt (Urban et al., 2015). However, the correlations between planting date and April temperature and precipitation, respectively, are hard to quantify, as temperature and precipitation at April are mostly negatively correlated in our study domain, except a small number of counties in the northwest (Figure 12F). These results may explain why April temperature has some correlation with corn yield (Figure 5). For the LowP.LowT group (north of 42N) lower April temperature delays planting (a negative correlation as shown in Figure 11B), however the late planting leads to a yield loss (a negative correlation shown in Figure 12C), thus actually a warmer April temperature leads to yield increase (Figure 5C). To the contrary, for most counties in the hot climate groups (south of 42N latitude), April temperature has a negative correlation with crop yield (Figures 5A,B), as lower April temperature in the south of the study domain delays planting (Figure 12D), and late planting leads to higher crop yield (Figure 12C). However, when 2012 data is excluded, earlier planting is found correlated with higher yield for counties south of 40N and in eastern Indiana (Figure 13).

#### Effect of Fertilizer Uses

Correlation analysis reveals that rainfed corn yield is not significantly correlated with nitrogen fertilizer use from the NUGIS source. For all the analyzed counties, the correlation coefficient between yield and nitrogen fertilizer rate is −0.21 (*p* < 0.01). This negative correlation is counter-intuitive, and may be due to the data uncertainty in the NUGIS data as well as the over-application of fertilizer; the latter may lead to the decoupling between the amount of fertilizer and crop yield. In addition, adding nitrogen fertilizer to the inputs does not increase the predictive accuracy of the data-driven models (see more details in section Machine Learning Models and In-season Predictability of Corn Yield). Given the indeterminate results and considering the uncertainty associated with NUGIS nitrogen fertilizer dataset, we decided not to include nitrogen fertilizer in the inputs of the final random forest data-driven models.

### Machine Learning Models and In-season Predictability of Corn Yield

**Figure 14:**
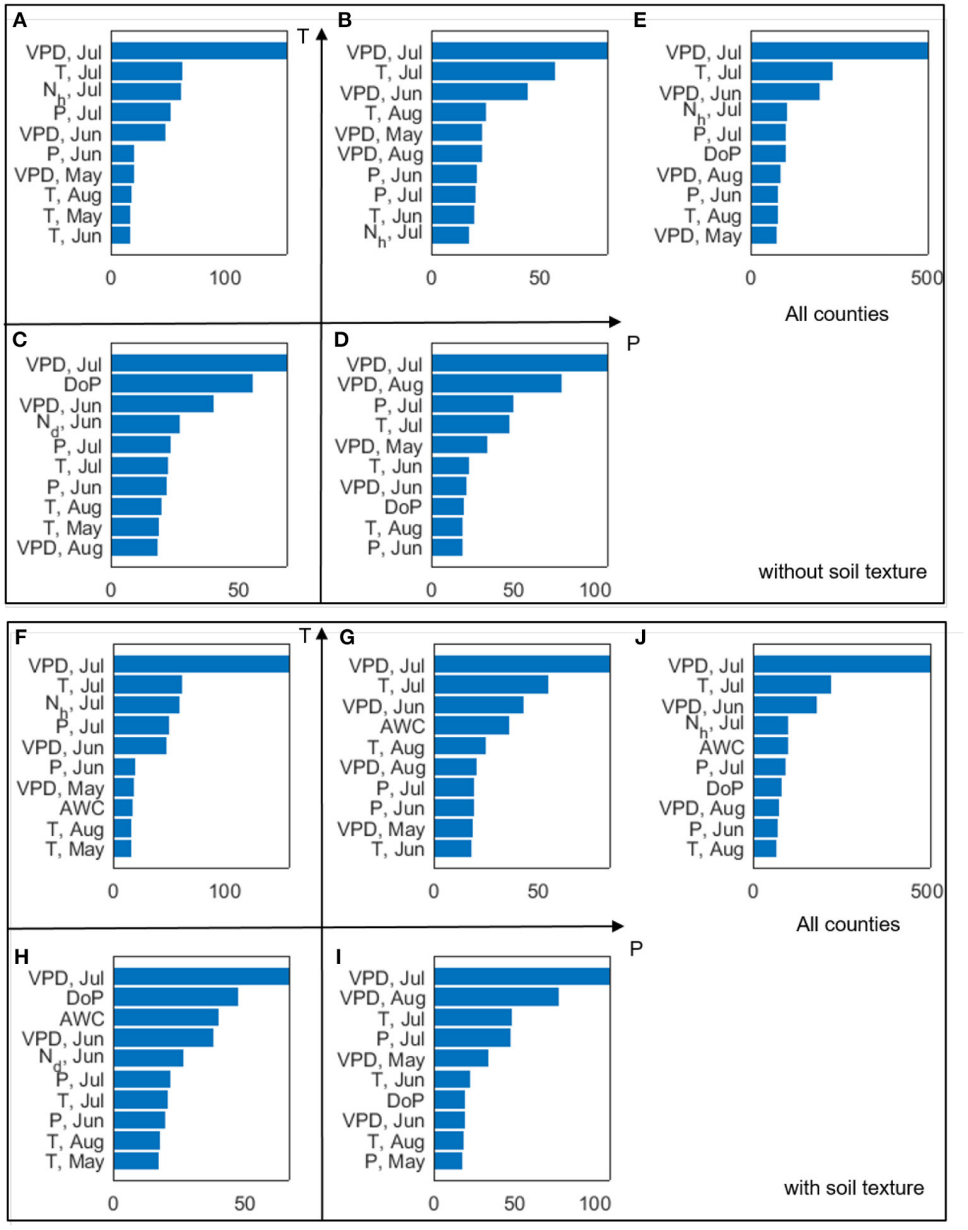
The 10 most important climate variables selected by the RF algorithm for each climate type **(A–D, F–I)** and all analyzed counties **(E,J)**. The horizontal axes indicate the variable importance. The top and bottom panels show results when (1) climate data and planting date are considered, and (2) climate data, planting date, and soil texture are considered, respectively.

The above analyses focus on the relationships between individual variables and corn yield. We then use the two machine learning algorithms (RF and LASSO) to combine all the potential predictors to predict end-of-season corn yield. We treat each county × year yield observation as an independent sample, and do not differentiate variabilities between space and time. Based on the trained RF model, we also calculate the variance importance of all the input variables and identify accordingly the most important predictors of yield. Figure 14 shows the top 10 most important variables when the inputs of the RF models include climate variables and planting date (Figures 14A–E), and when the inputs also include soil properties (Figures 14F–J).

**Figure 15:**
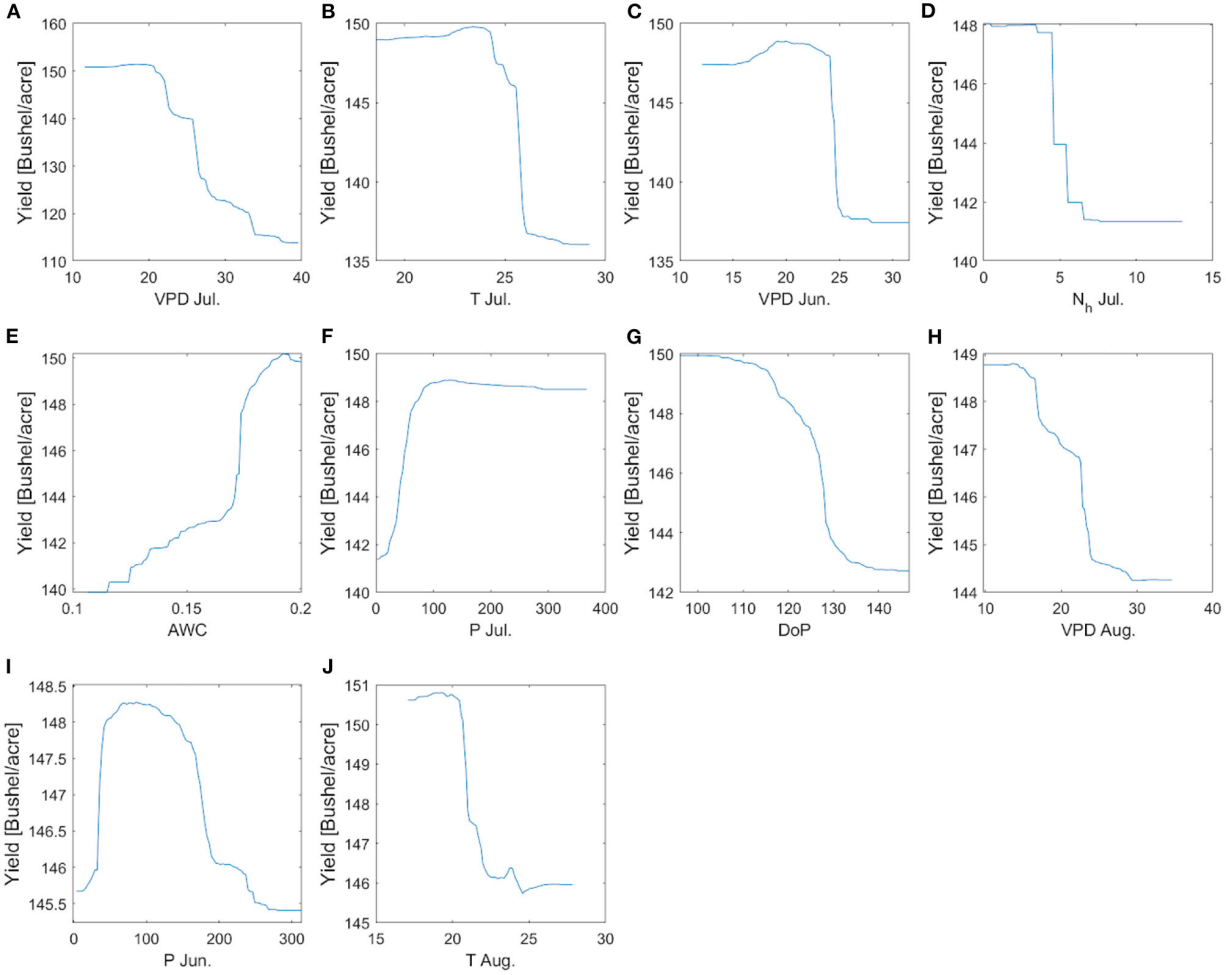
The partial dependence plots of yield with respect to the 10 most important predictors identified by RF when all counties are used. **(A–J)** is the plot corresponding to 10 features which can be read directly from axis.

We find that July VPD ranks as the most important predictor of corn yield for all the climate groups (Figure 14), which is consistent with our earlier finding (Figures 7–9) and prior literature (Lobell et al., 2014). Climate variables having high importance scores are mostly from VPD, T, N_h_, and P, and from July, with some from June or August. As for May climate variables, only VPD_May_ and T_May_ rank among the top 10. Planting date has been identified as important in the colder climate groups. When soil properties are included in the analysis (Figures 14F–J), AWC is chosen for three climate groups (Figures 14F–H) and also by the general model (Figure 14J). For the HighP.LowT climate group, AWC is not among the 10 most important predictors mainly because the spatial variability of AWC is low in this group (Supplementary Figure 8). Figure 15 shows the partial dependence plots for the 10 most important input variables generated by the RF model trained using data of all counties. The sensitivity of yield with respect to these inputs based on the RF model is largely consistent with our findings from correlation analyses (section Temporally Varying Correlation Patterns Between Climate Variables and Corn Yield Across the Corn Belt).

**Figure 16:**
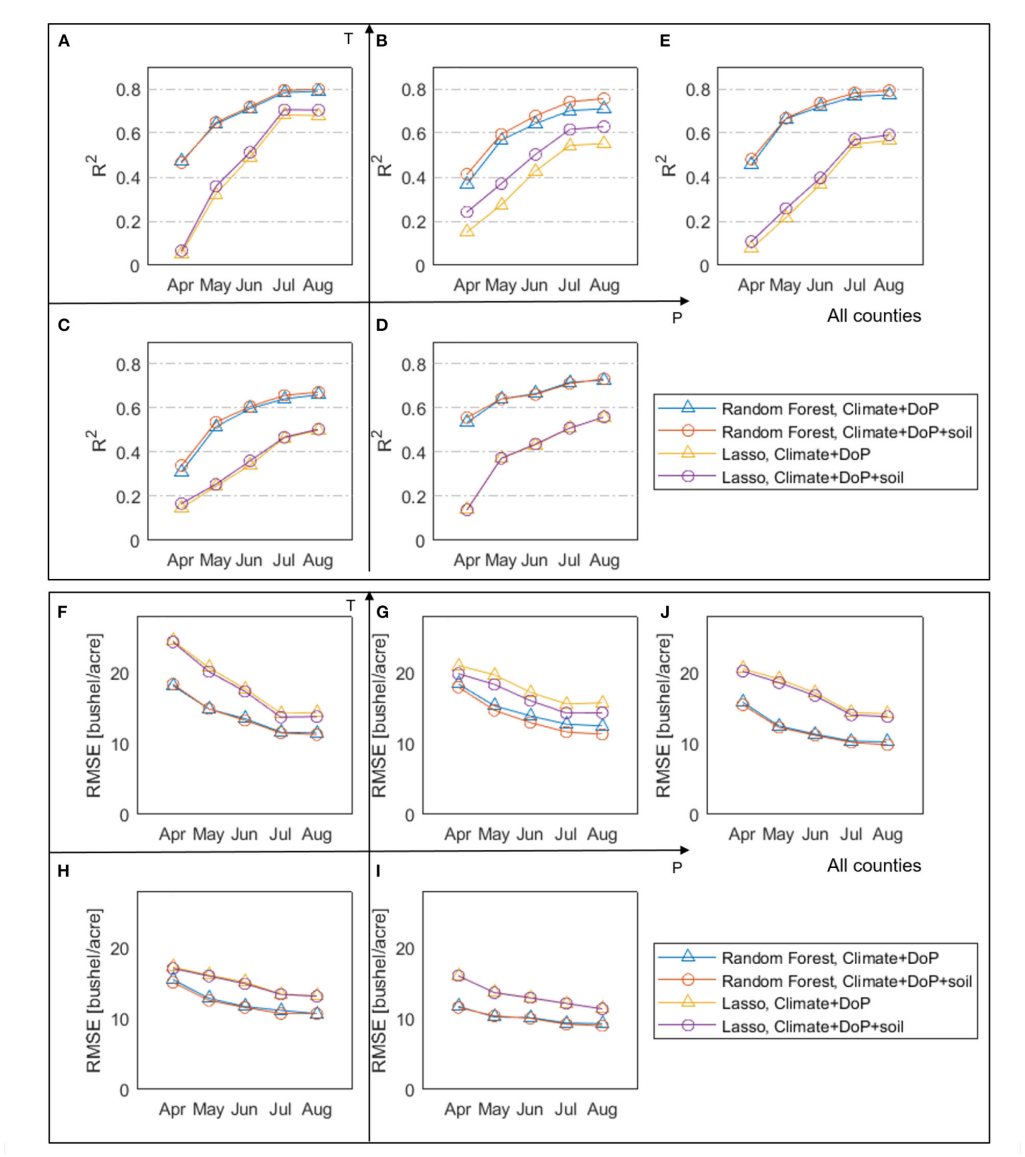
The coefficient of determination (*R*^2^) (top) and root-mean-square error (RMSE) (bottom) of the RF and LASSO models using as inputs climate and planting date data (blue) and climate, planting date and soil texture data (red). *R*^2^ is calculated for four groups **(A–D,F–I)** and all analyzed counties **(E,J)** based on a testing dataset that is not used for training the RF models.

Furthermore, we investigate the predictability of annual yield given the explanatory variables (i.e., planting date, soil texture, and climate data) using the RF and LASSO models. Figure 16 shows the model prediction performance on the testing data and how the prediction performance (measured by *R*^2^ and RMSE) changes with the target month (i.e., the month when prediction is made). For example, the *R*^2^ and RMSE in June correspond to the RF or LASSO model trained using time-invariant data (i.e., planting date, soil texture) and climate data from April to June. It thus should be expected that the yield predictability increases as the growing season progresses and more months of data become available.

We find that the RF model outperforms LASSO in all cases, where RF has higher *R*^2^ (0.66~0.80 vs. 0.50~0.70) and lower RMSE (8.9~12.5 vs. 11.4~15.4 bushels/acre) than LASSO for the four climate groups (Figure 16). Based on the *R*^2^ metric, Figure 16 shows that the highest prediction accuracy is achieved in the LowP.HighT group (*R*^2^ = 0.80), while the lowest accuracy is achieved in the HighP.LowT group (*R*^2^ = 0.67). In general, the addition of soil properties to the inputs slightly increases the predictive accuracy (*R*^2^ increased by 0.005~0.045 and RMSE reduces by 0~1.4 buchels/acre), compared to the prediction using only climate data and planting date. The HighP.HighT group has the most noticeable increase in *R*^2^, consistent with Figure 14G showing the relative importance of AWC.

## Discussions

### Corn Yield Response to Climate Variables Vary in Growing Season and Across the Corn Belt

Our results confirm a time-varying response of corn yield to climate variables. This time-varying response of corn yield is primarily because crop at different phenological stages have varying vulnerability to environmental stresses (Prasad et al., 2017; Peng et al., 2018). In other words, same degree of environmental stress (e.g., lack of rainfall, or heatwave) occurring at different times can lead to different consequences for corn yield. For four out of the five climate metrics (i.e., precipitation, temperature, heatwave, and VPD) that are the primary predictors (section Feature Selection and Correlation Analyses Between Climate Variables and Yield), correlation with corn yield in general increases with time since planting, peaks in July, and decreases in August (Figures 3, 5–7). This finding is consistent with the general understanding that July (flowering time) is the most critical period to affect corn yield in the U.S. Corn Belt, as the flower stage mostly happens in July for the study area. Flower stage of corn largely determines the grain number and thus the potentials of final yield and is sensitive to various environmental stresses (Çakir, 2004; Rattalino Edreira and Otegui, 2012). We also found that the number of dry days (N_d_) follows the similar trend but has the highest correlation in June (Figure 4), which may indicate that dry-spells have its largest damage to corn at the late vegetative stage (Çakir, 2004). Whereas, for precipitation and temperature, the magnitudes of correlation between the climate metrics and yield significantly drops after July to similar levels as in June (Figures 3, 5), heatwave and VPD show only a slight decrease in the correlation magnitude in August compared with in July, and still remain higher than June (Figures 6, 7). These patterns are consistent with findings in previous studies that heat stress, as expressed by the above two climate metrics, affects the later reproductive stage associated with the grain-filling (Rattalino Edreira et al., 2011, 2014). The above analysis can be compromised if there are strong auto-correlations between different months for the same climate metrics. However, auto-correlations beyond one-month lag are weak for all climate metrics used (Supplementary Figure 11).

In addition, our results reveal that the above temporally varying responses vary in space. For the four out of the five climate metrics (precipitation, temperature, heatwave, and VPD), the correlation with yield in July is the strongest in the LowP.HighT group (Figures 3A, 5A, 6A, 7A). This is largely because the Low P and High T conditions combined amplify the stress conditions. In particular, a dry condition (i.e., lower precipitation) limits the soil moisture supply, which leads to plant water stress; meanwhile, higher temperature further increases VPD and reduces the stomata conductance (Farquhar and Sharkey, 1982), leading to a further reduction of photosynthesis and over-depletion of soil moisture (Lobell et al., 2013). This finding thus confirms the deteriorating effects of combined drought and heat stress on ecosystem functioning (Law, 2014) and crop yield (Jin et al., 2016; Zampieri et al., 2017). Meanwhile, the HighP.LowT group usually delays the peak of correlation from July to August. This is largely because colder (northern) regions tend to have later planting dates, which in many cases means a later flower stage. Therefore, the delayed peak correlation pattern is mostly attributed to the delay of the most sensitive stage for corn growth.

### Disentangling Effects of VPD and Temperature on Corn Yield

As discussed in the introduction, the effects of temperature and VPD on corn yield have been under debate. The high correlation between these two climate variables makes it challenging to disentangle their individual contributions. In the current work, we provide a new and parsimonious way to decompose their contributions (section Disentangling the Confounding Effects of Temperature and VPD on Yield). In this analysis we only related yield to July VPD and July temperature because our results in sections Temporally Varying Correlation Patterns Between Climate Variables and Corn Yield Across the Corn Belt and Machine Learning Models and In-season Predictability of Corn Yield justify that July is in general the most critical month for determining corn yield for the U.S. Corn Belt. Our results show that both VPD and T play critical roles on corn yield, but the yield loss is mostly due to the high VPD (Figures 8, 9).

We found that the VPD effect on corn yield is predominantly negative, which is consistent with the understanding of the plant physiological processes (Lobell et al., 2014). In particular, VPD's effect is primarily through reducing the stomatal opening in a higher VPD environment (Farquhar and Sharkey, 1982; Katul et al., 2012). In the study area, this is likely the dominant pathway to reduce stomatal conductance. The complementary pathway, i.e., closing the stomata due to soil moisture deficit, occurs less frequently in the study area (Lobell et al., 2014), because otherwise irrigation would have been extensively adopted by farmers similarly as in much of Nebraska. The importance of VPD has also been confirmed by the machine learning-based modeling results that VPD in July is identified as the most important predictor rather than T or other climate variables.

While increasing VPD almost always negatively affects yield, the direct T effects is non-monotonic. As T increases, yield first increases and then decreases. The T value at the turning point indicates the optimal temperature for plant productivity. Equation (1) and Figure 8 suggests an optimal temperature in the range of 21–28°C, consistent with prior studies (Sage and Kubien, 2007; Hatfield and Prueger, 2015).

### Effects of Soil Properties and Managements on Corn Yield

Our study shows a strong correlation between higher AWC (and higher SOM) and enhanced corn yield, which confirms the findings from previous studies that soil properties (e.g., AWC, SOM) may improve corn yield in general (Kravchenko and Bullock, 2000). Besides, we found that higher SOM corresponds to lower yield inter-annual variability, indicating higher SOM may provide the buffering ability for yield variability and thus increase field-level yield resilience. However, in the RF model AWC receives high importance score whereas SOM is not among important variables. This is consistent with the fact that AWC has a higher positive correlation with yield (ρ = 0.53) than SOM (ρ = 0.30).

Agricultural management data (i.e., planting date and fertilizer rate) are usually difficult to find in the public domain especially for analyses at the regional scale. Two valuable datasets of planting date [Lobell et al., 2014] and fertilizer rate (International Plant Nutrition Institute (IPNI), 2011) enabled us to analyze the effects of management practices on yield in this study. As described in section Effects of Management on Corn Yield, planting date has a statistically significant correlation with yield, while nitrogen fertilizer rate does not. A likely reason is the potential uncertainty in the fertilizer sales data and its assumption that locally sold fertilizer is used locally. In addition, as farmers in the U.S. Corn Belt tend to apply more nitrogen than needed by crops (Vitousek et al., 2009), yield may be not sensitive to N rate variability in the study area. On the other hand, planting date is significantly correlated with both T and P, though with opposite signs. Specifically, planting date is negatively correlated with T, meaning higher T may lead to earlier planting. Meanwhile, planting date is positively correlated with P, suggesting that more P (wetter condition) may postpone planting. The above empirical findings are consistent with anecdotal knowledge from farmers and in literature (Waha et al., 2012; Urban et al., 2015) that farmers' planting decision is contingent on T and P.

Furthermore, we found that planting date has a spatially varying relationship with yield, with the northern part of the study area showing that late planting is related to lower yield, while most of the southern part showing that late planting is related to higher yield. The primary reason is that the planting date varies with local climate conditions, and together they determine the length of growing season and the degree of heat stress during the peak growing season. We further found that planting date has been identified among the top 10 predictors by the RF model, suggesting that the inclusion of such information is conducive for yield modeling (section Machine Learning Approach and Its Prediction Performance). Despite the importance of planting date, spatially explicit planting date information is not available in the public domain, and USDA only provides region-aggregated crop progress report at the Agricultural District level (~aggregating 8–15 counties) and at state level. Satellite remote sensing may provide a solution to obtain spatially explicit planting date information (Urban et al., 2018). With the emergence of more high-resolution satellite data (Houborg and McCabe, 2018), it is likely that field-level planting date estimation will be available in the near term.

### Machine Learning Approach and Its Prediction Performance

According to the RF model, the three climate variables, namely, VPD, T, and P, during the peak growing season (July, Jun, Aug) are the most important predictors. The two climate extreme metrics, number of dry days (N_d_) and heatwave (N_h_) in July also receives high importance scores, suggesting the critical role of heat stress in determining end-of-season corn yield; this effect may not be captured found that planting date and AWC have been identified by RF among the top 10 predictors for corn yield, confirming the importance of management practices and soil properties in determining corn yield. The most important inputs identified each climate group or all counties are used to train the models. This again highlights the spatial patterns of yield responses.

It is worth noting that our current modeling framework treats each county×year as an independent sample and models spatio-temporal variabilities together rather than differentiating them. This approach is different from the “panel models” in econometrics (Lobell and Burke, 2010), which, if applied in our context, would have a fixed effect for each county (indicating time-invariant feature that may be caused by county-specific soil properties and/or management practices) and all the counties share the same response function of corn yield with climate variables. While providing a way to disaggregate yield response to temporally varying and invariant factors, due to its linear regression nature panel regression may fall short for accurate modeling and prediction of spatiotemporal variability of yield resulting from the coupled controls of climate, soil, and management factors.

As of in-season prediction of rainfed corn yield, the RF models outperform LASSO models in all climate groups (Figure 16). The main reason is that the linear LASSO model does not account for the non-linearity of yield response to predictors or interaction among predictors, while RF is a non-parametric approach capable of learning complex, non-linear relations between yield and predictors from data without the need to explicitly model them. Although additional terms can be built into the LASSO model to represent prescribed non-linearity and interaction, this would result in a large number of input variables and, more importantly, deviate from the motivation of this study of objectively inferring relationships from data without prescribed parameterization. In addition, due to the *L*_1_ regularization, LASSO sets coefficients of 21~40% (varying among climate groups) predictors to zero, resulting in parsimonious models. In particular, when two or more predictors are correlated (i.e., collinearity), LASSO tends to set the coefficients but one of these predictors to zero. We found that for all climate groups, LASSO selected July VPD among the 10 most important variables, but set July T coefficient to zero. This is not surprising, because July T and VPD are correlated and yield responds monotonically to VPD but non-linearly to T. As discussed in section Disentangling Effects of VPD and Temperature on Corn Yield, July VPD and T may affect yield through different mechanisms. In this case, collinear predictors are not necessarily redundant (Dormann et al., 2013). Thus, including both predictors may be desirable for physical interpretability and prediction performance. In contrast to LASSO, RF tends to retain collinear predictors due to its random candidate subset for splitting variables. As shown in Figure 14, July VPD and T are both selected by RF among the most important variables in all cases.

For both models, as we progress further into the growing season with more available climate data ingested in, the model performance increases. Averaged over all climate groups, the RF model reaches a high accuracy of *R*^2^ = 0.792 by the end of August to predict the concurrent year's final corn yield, and the same model has achieved *R*^2^ = 0.781 by the end of July. These results indicate a promising capability of using the machine learning-based models to predict county-level corn yield at regional to continental scales.

## Conclusions

We performed a data-driven analysis on rainfed corn yield in the U.S. Corn Belt at the county level for the study period of 2000–2012 to objectively infer how yield responds to climate, soil properties, and management practices at the regional scale. Our results confirmed previous findings as well as led to enhanced quantitative understanding of the controlling factors on corn yield. Correlation analyses revealed predominant effects of climate average and extreme conditions on rainfed corn yield. Our results confirmed that corn yield response to climate variables change through crop development stages, and further showed that the temporally varying responses vary in space as controlled by regional climate. We then use regression analysis to decompose the effects of temperature and VPD. Increasing VPD leads to monotonically decreasing yield, while temperature effects is non-monotonic. In addition to climate variables, soil properties and management practices contribute to the spatiotemporal variability of rainfed corn yield. More specifically, our results showed that higher AWC enhances yield, consistent with findings from previous studies (e.g., Kravchenko and Bullock, 2000), while higher SOM corresponds to lower yield inter-annual variability. Planting date has a statistically significant correlation with yield, and that planting date is affected with early season climate conditions. No significant correlation is found between nitrogen fertilizer application rate and yield. Lastly, we used two machine learning algorithms (random forest and LASSO) to model rainfed corn yield using the climate, soil, and management practice variables as predictors. RF model outperformed LASSO for in-season prediction skill and achieved a high accuracy by the end of July. The promising results suggest machine learning as an efficient and skillful approach for regional scale prediction of crop yields.

## Data Availability Statement

Data and codes used in this study are available at https://www.hydroshare.org/resource/3a80e06f59784a93a4e2acabf8a3ec93/.

## Author Contributions

TX designed and conducted the analyses. KG initiated this work and co-designed experiments. KG and BP contributed to the data preparation. All authors contributed to the writing of this manuscript.

## Conflict of Interest

The authors declare that the research was conducted in the absence of any commercial or financial relationships that could be construed as a potential conflict of interest.
