# Supplementary material for: Machine Learning-Based Modeling of Spatio-Temporally Varying Responses of Rainfed Corn Yield to Climate, Soil, and Management in the U.S. Corn Belt
Source: Front Artif Intell. 2021 May 28;4:647999. doi: 10.3389/frai.2021.647999 (PMC8192978; doi:10.3389/frai.2021.647999)
Supplement: Supplementary file 1 [file Data_Sheet_1.docx]

Supplementary Material


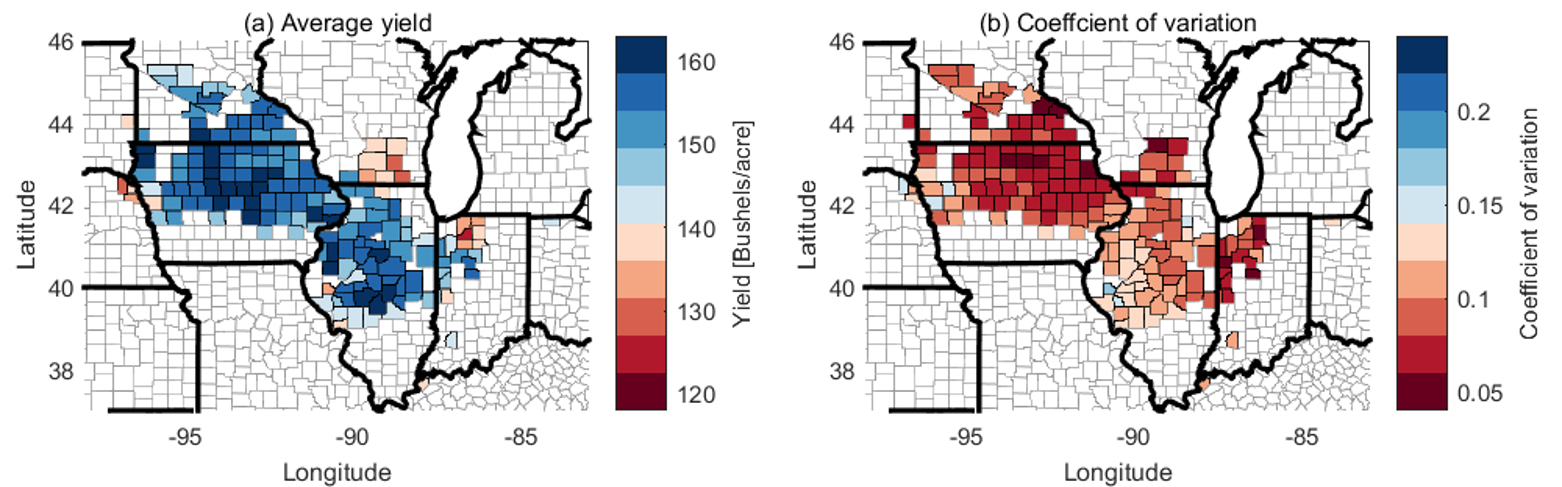


Figure S1. The multi-year average yield and inter-annual coefficient of variation (C.V.), evaluated during 2000-2011.


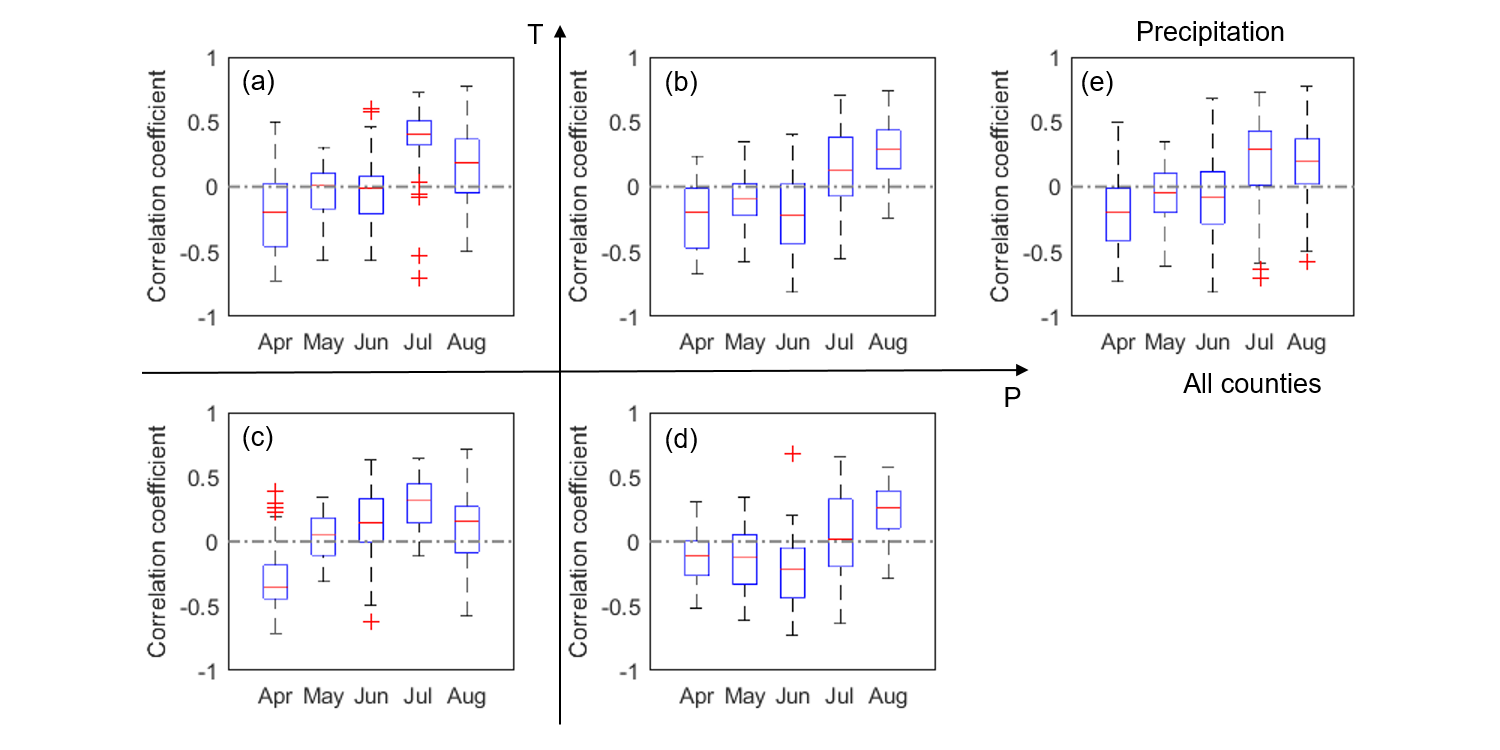


Figure S2. Box plots of correlation coefficient ($r$) between corn yield and monthly total precipitation, P, evaluated for four groups (a-d) and all analyzed counties (e) in 2000-2012. The horizontal and vertical axes indicate long-term average precipitation and temperature, corresponding to the axes in Figure 2(b).


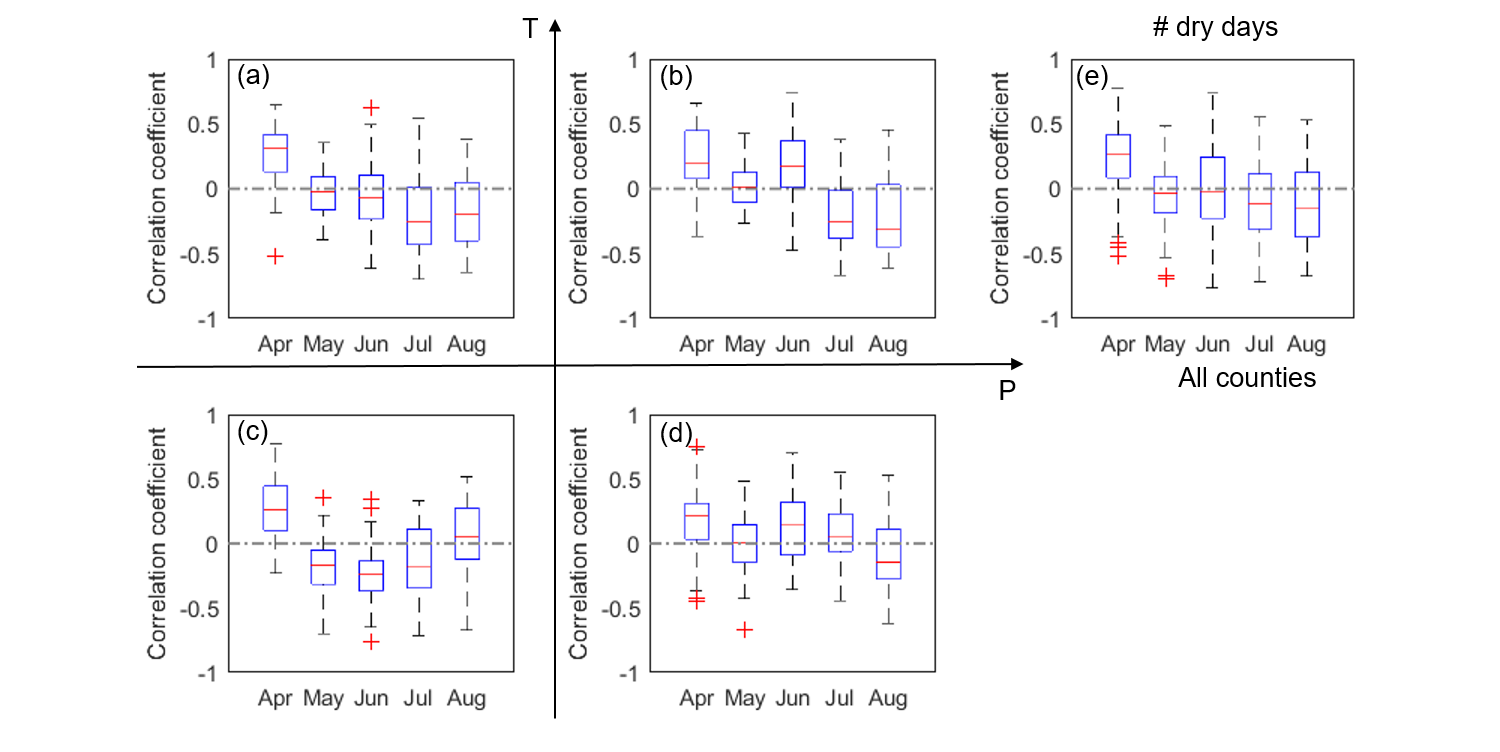


Figure S3. Box plots of correlation coefficient ($r$) between corn yield and number of dry days, N_d_, evaluated for four climate groups (a-d) and all analyzed counties (e) in 2000-2012.


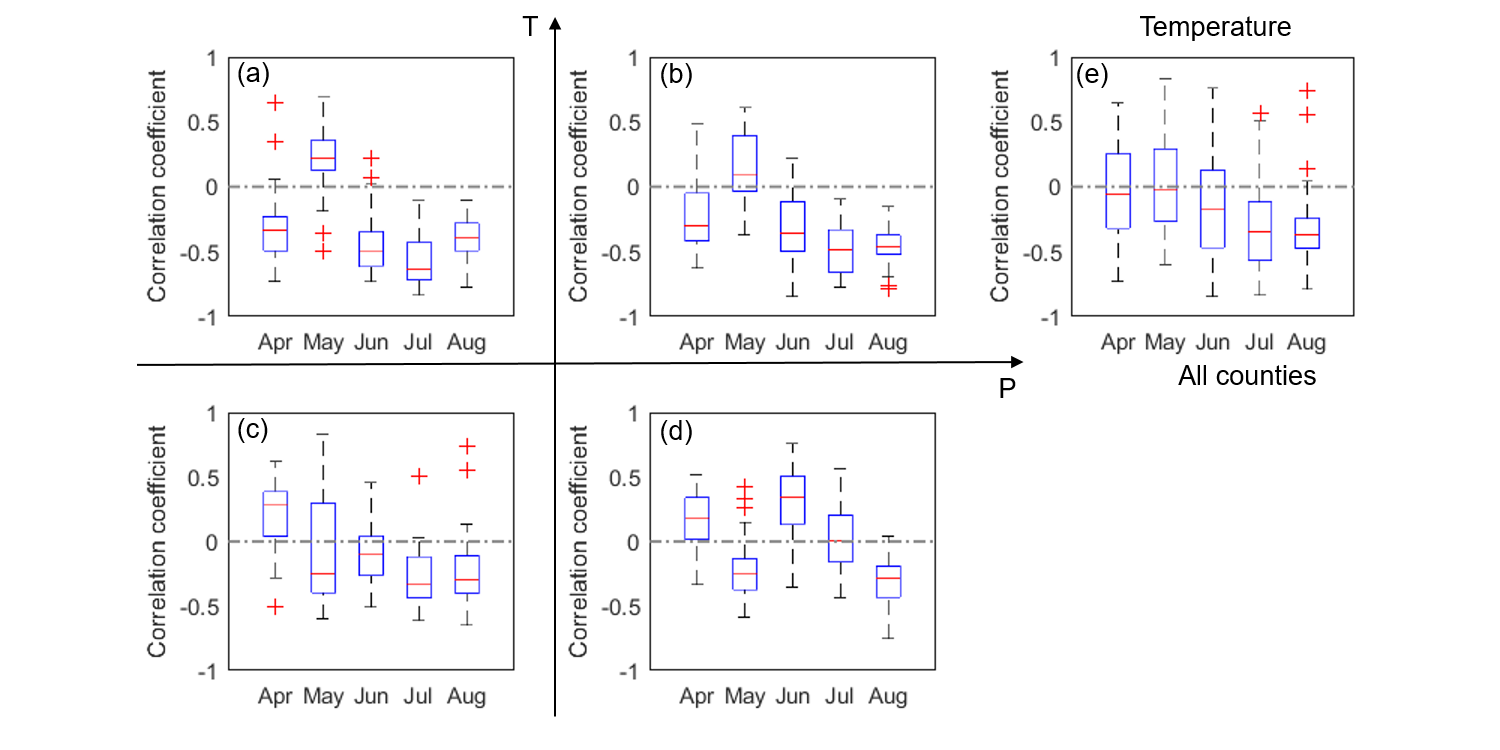


Figure S4. Box plots of correlation coefficient ($r$) between corn yield and monthly mean temperature, T, evaluated for four groups (a-d) and all analyzed counties (e) in 2000-2012.


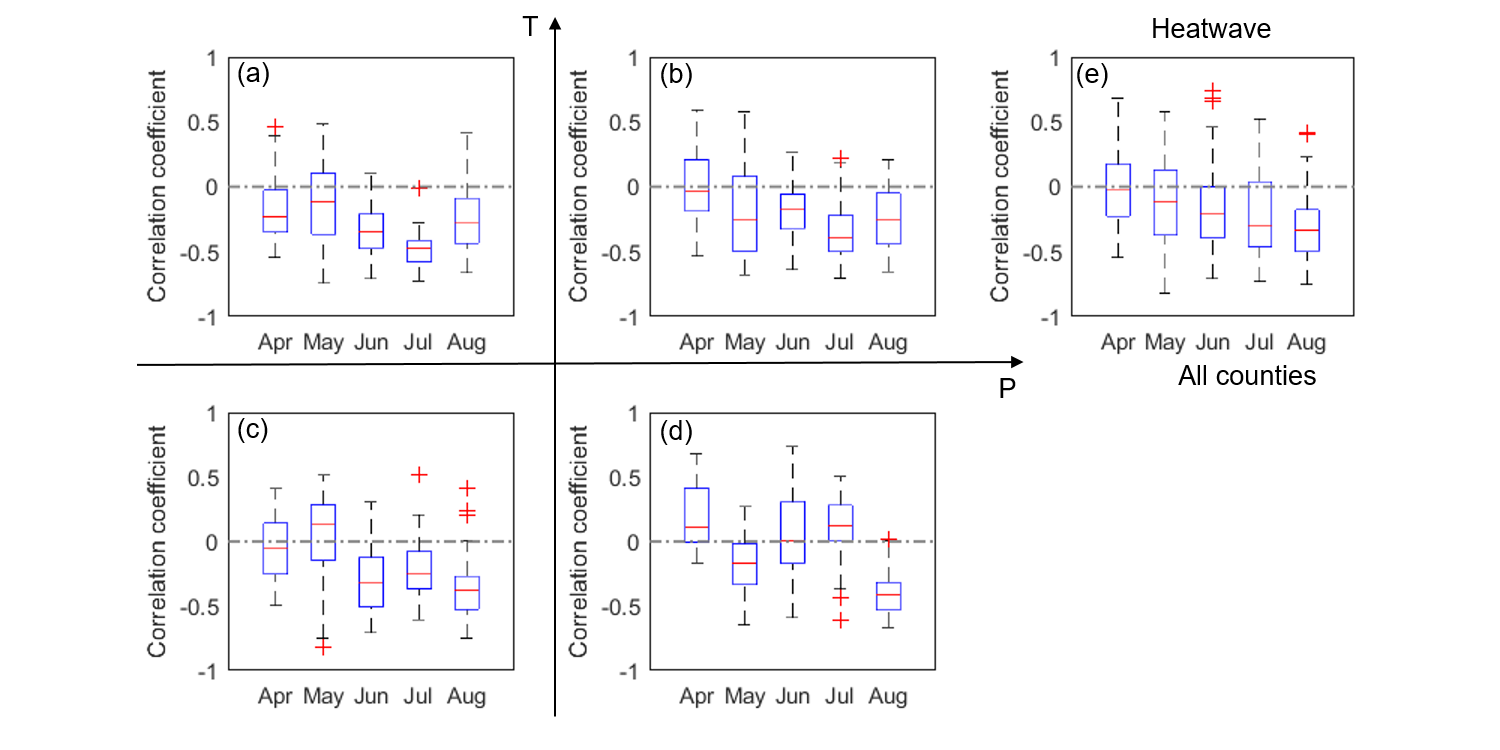


Figure S5. Box plots of correlation coefficient ($r$) between maize yield and heatwave, N_h_, evaluated for four groups (a-d) and all analyzed counties (e) in 2000-2012.


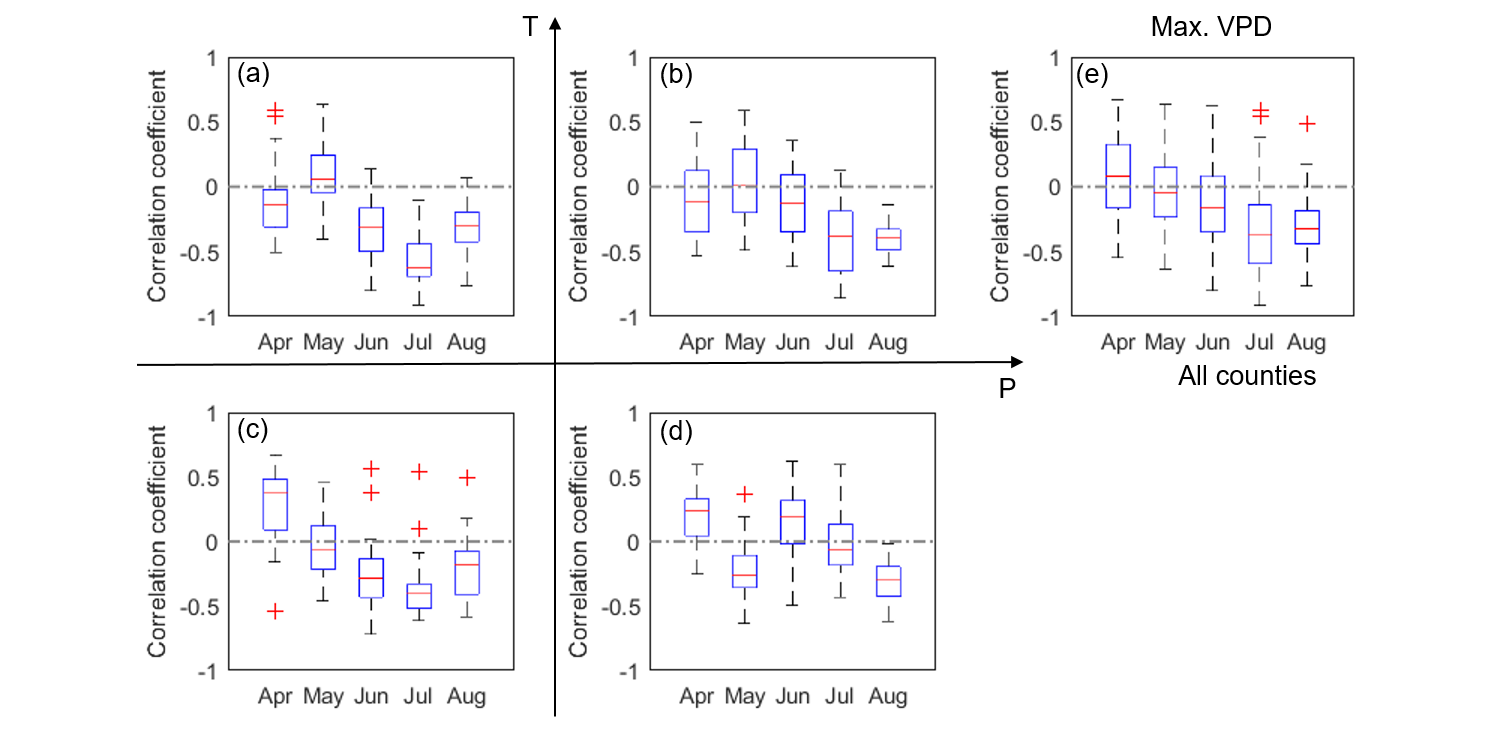


Figure S6. Box plots of correlation coefficient ($r$) between corn yield and VPD, evaluated for four groups (a-d) and all analyzed counties (e) in 2000-2012.


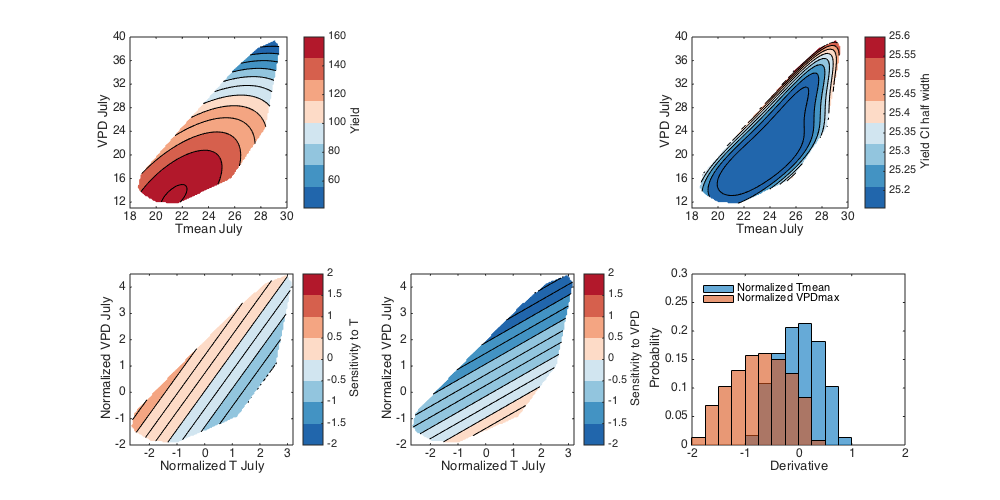


Figure S7. The contour plots of half width of 90% prediction interval of Equation (1) in main text.


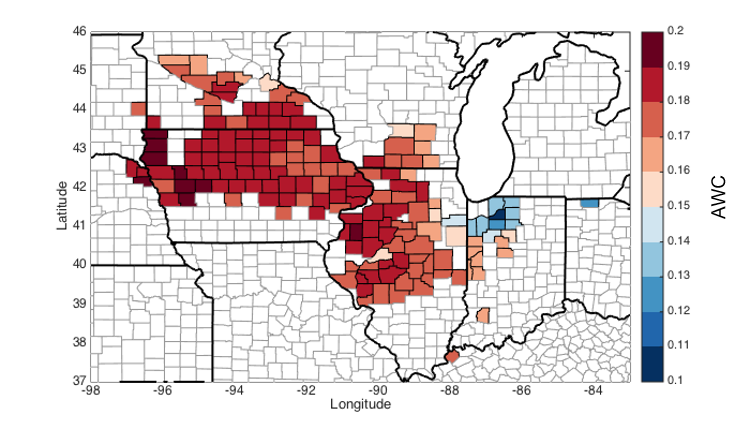


Figure S8. Plant-available soil water content (AWC) for 166 counties in the U.S. Corn Belt.


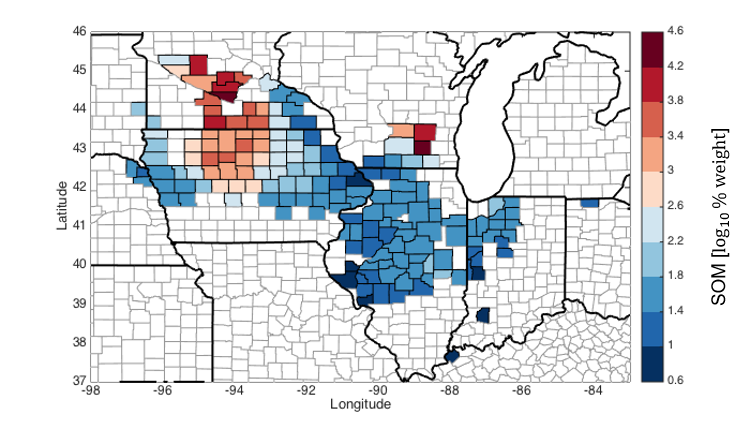


Figure S9. Soil organic matter (SOM) for 166 counties in the U.S. Corn Belt.


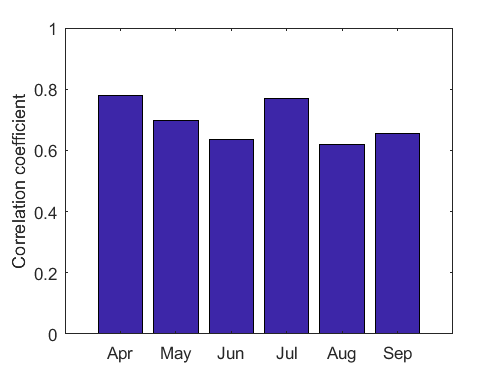


Figure S10. Correlation coefficient between monthly mean temperature and monthly mean daily maximum VPD from April to September.


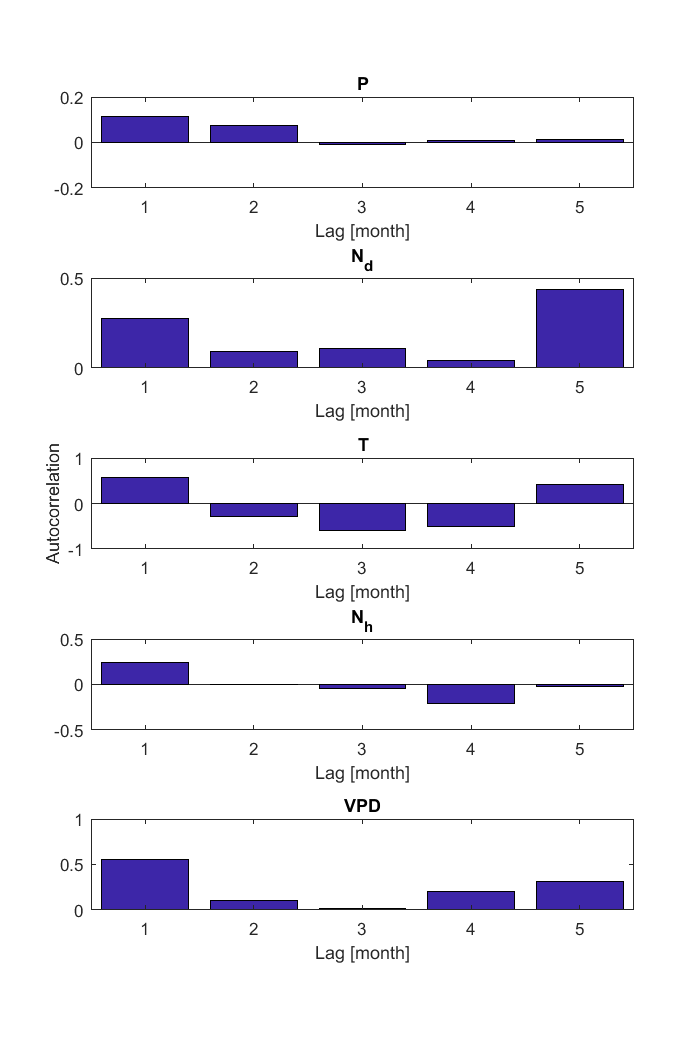


Figure S11. Autocorrelation of climatic variables, namely monthly total precipitation, number of dry days, mean temperature, heatwave and mean daily maximum VPD.


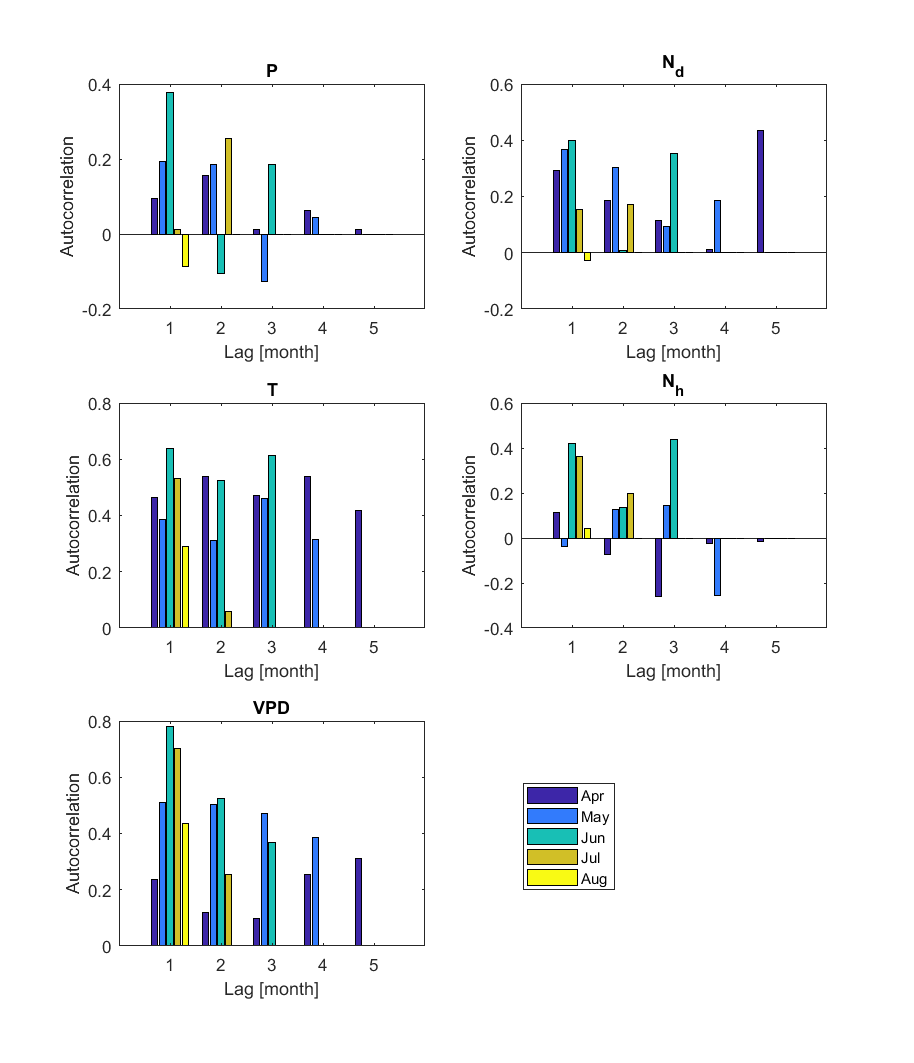


Figure S12. Autocorrelation calculated by climatic factor and month (color coded). For example, in panel P, the second blue bar shows the correlation coefficient between precipitation in April and May.
